# Impact of a Prior Cancer Diagnosis on Quality of Care and Survival Following Acute Myocardial Infarction: Retrospective Population-Based Cohort Study in England

**DOI:** 10.1161/CIRCOUTCOMES.122.009236

**Published:** 2023-06-20

**Authors:** Lucy Teece, Michael J. Sweeting, Marlous Hall, Briana Coles, Clare Oliver-Williams, Cathy A. Welch, Mark A. de Belder, John Deanfield, Clive Weston, Mark J. Rutherford, Lizz Paley, Umesh T. Kadam, Paul C. Lambert, Michael D. Peake, Chris P. Gale, David Adlam

**Affiliations:** Department of Health Sciences (L.T., M.J.S., B.C., C.O.-W., C.A.W., M.J.R., U.T.K., P.C.L.), University of Leicester, United Kingdom.; Department of Respiratory Medicine (M.D.P.), University of Leicester, United Kingdom.; Department of Cardiovascular Sciences and NIHR Leicester Biomedical Research Centre (D.A.), University of Leicester, United Kingdom.; National Cancer Registration and Analysis Service, NHS Digital, London, United Kingdom (L.T., M.J.S., B.C., C.O.-W., C.A.W., L.P., M.D.P.).; Leeds Institute of Cardiovascular and Metabolic Medicine, University of Leeds, United Kingdom (M.H., C.P.G.).; National Institute for Cardiovascular Outcomes Research, Barts Health NHS Trust, London, United Kingdom (M.A.d.B., J.D., C.W.).; Institute of Cardiovascular Science, University College London, United Kingdom (J.D.).; Department of Cardiology, Glangwili General Hospital, Carmarthen, United Kingdom (C.W.).; Leicester Diabetes Centre, United Kingdom (U.T.K.).; Department of Medical Epidemiology and Biostatistics, Karolinska Institutet, Stockholm, Sweden (P.C.L.).

**Keywords:** humans, myocardial infarction, neoplasms, prognosis, retrospective studies

## Abstract

An increasing proportion of patients with cancer experience acute myocardial infarction (AMI). We investigated differences in quality of AMI care and survival between patients with and without previous cancer diagnoses.

**Methods:**

A retrospective cohort study using Virtual Cardio-Oncology Research Initiative data. Patients aged 40+ years hospitalized in England with AMI between January 2010 and March 2018 were assessed, ascertaining previous cancers diagnosed within 15 years. Multivariable regression was used to assess effects of cancer diagnosis, time, stage, and site on international quality indicators and mortality.

**Results:**

Of 512 388 patients with AMI (mean age, 69.3 years; 33.5% women), 42 187 (8.2%) had previous cancers. Patients with cancer had significantly lower use of ACE (angiotensin-converting enzyme) inhibitors/angiotensin receptor blockers (mean percentage point decrease [mppd], 2.6% [95% CI, 1.8–3.4]) and lower overall composite care (mppd, 1.2% [95% CI, 0.9–1.6]). Poorer quality indicator attainment was observed in patients with cancer diagnosed in the last year (mppd, 1.4% [95% CI, 1.8–1.0]), with later stage disease (mppd, 2.5% [95% CI, 3.3–1.4]), and with lung cancer (mppd, 2.2% [95% CI, 3.0–1.3]). Twelve-month all-cause survival was 90.5% in noncancer controls and 86.3% in adjusted counterfactual controls. Differences in post-AMI survival were driven by cancer-related deaths. Modeling improving quality indicator attainment to noncancer patient levels showed modest 12-month survival benefits (lung cancer, 0.6%; other cancers, 0.3%).

**Conclusions:**

Measures of quality of AMI care are poorer in patients with cancer, with lower use of secondary prevention medications. Findings are primarily driven by differences in age and comorbidities between cancer and noncancer populations and attenuated after adjustment. The largest impact was observed in recent cancer diagnoses (<1 year) and lung cancer. Further investigation will determine whether differences reflect appropriate management according to cancer prognosis or whether opportunities to improve AMI outcomes in patients with cancer exist.

**What is Known**

- To date, studies have shown that those with previous cancer diagnoses presenting with acute myocardial infarctions are older and have more comorbidities than those without.
- Those with prior cancer are reportedly treated more conservatively following acute myocardial infarction and with lower usage of evidence-based medications compared with those without, although these findings may be confounded by differences between cancer and cancer-free populations.
- There are mixed findings as to the effect of cancer on rates of in-hospital complications and mortality rates but evidence of higher long-term mortality (mainly cancer related) and higher readmission rates.

**What the Study Adds**

- To our knowledge, this is the first national cardio-oncology study to investigate the impact of a prior cancer diagnosis on internationally recognized quality-of-care indicators for acute myocardial infarction.
- Using a potential-outcomes framework enables us to quantify the direct effects of previous cancer diagnoses on treatment following acute myocardial infarction, while adjusting for differences in patient characteristics.

**See Editorial by Quintana et al**

Heart disease and cancer remain the leading causes of mortality in developed health care economies.^1^ Survival is improving for most tumor sites, and, as cancer-specific outcomes improve, comorbidities will have an increasing impact on the outcomes of cancer survivors.^2^ Cancer and heart disease share common risk factors including age, smoking, hypertension, diabetes, and obesity. Furthermore, cancer treatments can themselves increase the risk of heart disease and cardiovascular events.^3,4^ Taken together, these factors mean an increasing number of patients with cancer will later develop heart disease.^5^

Outcomes following acute myocardial infarction (AMI) in patients with cancer are reported to be significantly worse than for noncancer patients with higher long-term mortality and readmission rates compared with noncancer patients.^6–9^ It is not known to what extent worse outcomes are avoidable. One hypothesis is that patients with cancer receive different treatment for AMI, due to clinical concerns about the appropriateness of standard guideline-based approaches in patients with a limited prognosis or who may be at increased risk, for example, of bleeding complications from antiplatelet therapies. There is some supportive evidence from observational studies of a reduced recourse to revascularization by percutaneous coronary intervention (PCI) and reduced use of drug-eluting stents in patients with cancer-AMI compared with noncancer patients.^8^ However, some or all of these differences may be influenced by confounding arising from differences in patient demographics (age, sex, and socioeconomic factors) and comorbidities and are likely to differ according to cancer-specific characteristics such as time since cancer diagnosis, cancer site, and cancer stage. As a result, evidence on whether the management of patients following AMI differs between cancer survivors and those without cancer remains inconclusive.

Measurement of the quality of overall AMI care is challenging due to the complexity of defining and quantifying the critical elements that constitute optimal care. To facilitate a consistent approach to such comparisons, the European Society of Cardiology has adopted standardized quality indicators (QIs) based on contemporary guideline-based recommendations for AMI management to allow benchmarking of AMI quality of care in different clinical contexts.^10,11^ These QIs have been shown to directly relate to important clinical outcomes, including an inverse association with 30-day mortality.^12^

The Virtual Cardio-Oncology Research Initiative (VICORI) has generated a nationwide data resource linking cancer and cardiovascular audit data with hospital coding and death certification data.^13^ This has provided a unique means to investigate quality of AMI care in patients with cancer at scale. We, therefore, aimed to use established AMI QIs to assess whether there is a disparity in care for AMI between patients with cancer and noncancer patients in England and to assess the impact of any differences on survival after AMI in this population.

## Methods

### Study Population

The study uses data from the VICORI, which brings together English national cancer data and 6 national cardiovascular disease (CVD) audits to enable investigation into the interplay between CVD and cancer.^13^

Specifically, a cohort of patients hospitalized with AMI was identified from the Myocardial Ischaemia National Audit Project (MINAP)—a comprehensive registry of acute coronary syndrome hospitalizations.^14,15^ Using a previously determined algorithm that combines information from discharge diagnosis, cardiovascular biomarkers, and electrocardiographic findings, we identified AMI hospitalizations and subdivided by phenotype into ST-segment–elevation MI (STEMI) and non-STEMI (NSTEMI^16^; Figure S1). Patients were included if aged ≥40 years and hospitalized in England between January 1, 2010, and March 31, 2018. The earliest hospitalization during the study period was used in the instance where patients had multiple admissions. Those with missing sex or National Health Service number were excluded as this obstructed linkage to other key data sets.

### Previous Cancer Diagnosis

The cohort was linked via a pseudonymized National Health Service number to the National Cancer Registration Dataset,^17^ held by the National Cancer Registration and Analysis Service, to determine previous cancer diagnoses. Data were extracted on cancer diagnoses, excluding noninvasive cancers or nonmelanoma skin cancers, within 15 years before the date of AMI hospitalization. Patients with no cancer diagnosis in the 15 years before the AMI admission were labeled as controls for the purposes of these analyses. Information for the latest tumor diagnosis was extracted for those patients with multiple past tumors.

Patient-level information, including demographics, medical history and comorbidities, admission biomarkers, and patient care (including treatment), was obtained from MINAP. Cancer-specific information including diagnosis date, tumor site, and stage at diagnosis was extracted from the linked National Cancer Registration Dataset, with cancer treatment information obtained from linked chemotherapy,^18^ radiotherapy,^19^ and hospital data. Vital status and date of death, though not specific cause of death, was identified through linkage of MINAP to the Office for National Statistics mortality data. Cause of death information was available through National Cancer Registration Dataset for patients with cancer only.

### Evidence-Based Hospital Treatment

Features of hospital treatment during admission for AMI were taken from the European Society of Cardiology QIs for the evaluation of care.^11^ MINAP was used to assess attainment for 13 of the QIs based on previous research that has mapped components of the QIs to relevant MINAP data items.^12,20^ Further information is given in the Supplemental Methods with the QIs described in Table S1.

For each QI, patients for whom treatment was deemed inappropriate, contraindicated, not applicable, or who had declined treatment (reported using MINAP data) were considered ineligible for assessment.

### Statistical Analysis

Baseline characteristics are reported stratified by cancer cases and controls and additionally assessed using an age-matched sample (1:5 ratio). We report the frequency and proportion of missing information and address this with multiple imputation with chained equations. Early management of patients is summarized according to AMI phenotype. Cancer-specific information is reported stratified by tumor site. The number of patients eligible for assessment is reported alongside the frequency and percent of QI attainment for cases and controls and unadjusted differences with 95% CIs.

We used a potential-outcomes framework to estimate the differences in QI attainment in those with cancer (compared with attainment had they not had cancer) after accounting for potential confounders via a series of multivariable regression models with robust SEs. An initial age-adjusted model was fitted, followed by a fully adjusted model that additionally included sex, AMI phenotype, comorbidities, smoking status, and previous CVD procedures. The effect modification of time since cancer diagnosis, cancer site, and cancer stage cancer was investigated. Assessment of statistically significant differences between cancer cases and controls in the primary analysis of 13 QIs was made using a Bonferroni-corrected threshold (*P*=0.05/13=0.0038). Sensitivity analyses clustering patients by hospital site and comparing results to other potential outcome approaches were conducted.

Survival up to a year post-hospital discharge was examined with flexible parametric survival models.^21^ Standardized postdischarge survival curves for patients with cancer and counterfactual controls are presented for all-cause and non–cancer-related mortality. The effect of suboptimal QI attainment on survival was explored using a mediation analysis,^20^ with QI attainment as the mediator between cancer (as the exposure) and survival (as the outcome). We obtained survival estimates for cancer cases had they received comparable levels of care as equivalent noncancer controls.

This study is reported in line with the Reporting of Studies Conducted Using Observational Routinely-Collected Health Data reporting guidelines.^22^ Further details of the statistical methods are given in the Supplemental Methods. This study was reviewed and approved by the VICORI Consortium Project Review Panel. The VICORI research programme has received favorable ethical opinion from the North East–Newcastle & North Tyneside 2 Research Ethics Committee (REC reference 18/NE/0123).

### Data Disclosure

Patient-level electronic health records obtained through VICORI can only be obtained by successfully applying for access to linked VICORI data by contacting vicori@le.ac.uk. An application for data access is subject to approval of a project proposal, analysis plan, and data request by the VICORI Project Review Panel and a formal application to the Office for Data Release at NHS Digital. The programming code and aggregate statistics that support the findings of this study are available from the corresponding author upon reasonable request.^35–39^

## Results

### Study Population

A cohort of 512 388 patients aged ≥40 years with first AMI hospitalization between January 2010 and March 2018 was identified from 209 hospitals in England from MINAP. Of the AMI cohort, 42 187 (8.2%) had a previous cancer diagnosis within 15 years before hospitalization. Based on the most recent cancer diagnosis, 11 498 (27%) were prostate, 6043 (14%) colorectal, 5236 (12%) breast, 2870 (7%) lung, and 16 540 (40%) were other diagnoses (Table S2). The majority of cancers were diagnosed at stages I and II with the exception of lung cancer where stages III and IV were more common. The median time from cancer diagnosis to AMI admission varied by site ranging from only 1 year for patients with lung cancer up to 6 years for patients with breast cancer. Between 3% (prostate) and 39% (bladder) of patients with cancer had received chemotherapy.

There were moderate amounts of missing data for baseline covariates, with values missing in ≈10% to 20% for most variables, while one-third of ethnicity and family history of coronary heart disease data were missing. Missingness was similar across cancer cases and noncancer controls (Table S3). Age, sex, and AMI phenotype were complete for the whole cohort as these variables were required for identification and linkage across data sets.

At the time of admission with AMI, cancer cases were on average 7 years older than the noncancer controls (mean age, 76.2 versus 69.2 years, respectively), were more likely to have an NSTEMI presentation, had generally a higher prevalence of comorbidities, were more likely to be ex-smokers (and less likely to be current smokers), and were more likely to be on CVD medications before admission (Table 1).

**Table 1.**
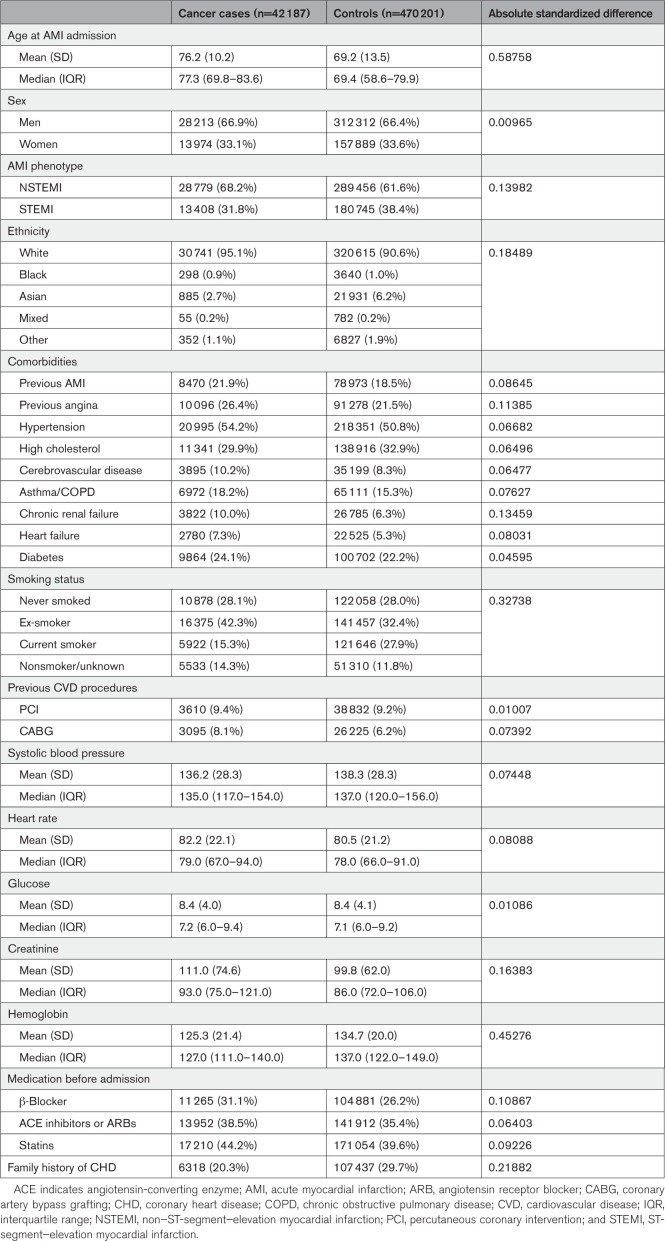
Baseline Characteristics of Patients Hospitalized With AMI, According to Cancer History

After accounting for age differences using age-matched controls, differences in comorbidity prevalence were similar for AMI, angina, heart failure, and diabetes, but differences remained for other comorbidities (Table S4). Differences in prior CVD medication use between cases and age-matched controls were less marked than in the original (not age matched) cohort.

### In-Hospital Treatments

Cancer cases were less likely to receive angiography or revascularization compared with noncancer controls regardless of AMI phenotype (Table 2). In STEMI presentations, cancer cases were less likely to receive an angiogram (82% versus 90%), PCI (72% versus 81%), or a coronary artery bypass grafting (0.9% versus 1.5%) compared with STEMI noncancer controls. Similarly in NSTEMI, cancer cases were less likely to receive an angiogram (55% versus 68%), PCI (26% versus 34%), or a coronary artery bypass grafting (3.0% versus 4.2%; Table 2). These differences were attenuated, yet remained, in age-matched comparisons (Table S5).

**Table 2.**
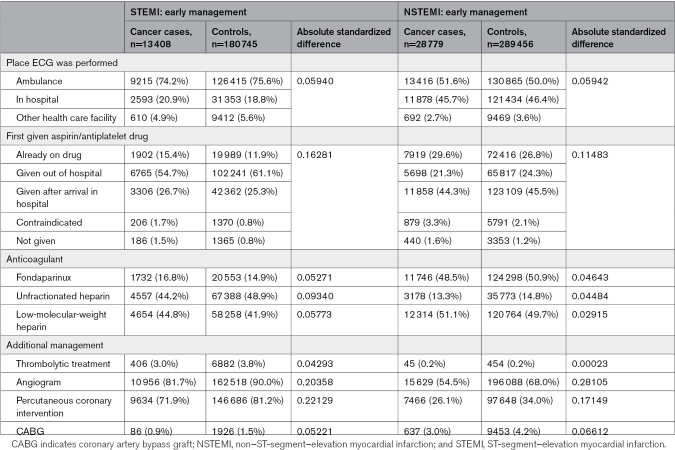
Early Management and In-Hospital Treatment of Patients Admitted for Acute Myocardial Infarction, According to Cancer History

### Assessment and Attainment of QIs of AMI Care

Recording and attainment of recognized AMI QIs were used to provide a structured assessment of quality of key components of AMI care. Patients with cancer were more often deemed ineligible for the assessment of a QI or were more often missing information used to assess eligibility than age-matched controls (Table S6). Attainment of QIs was generally high for both cancer cases and controls, with many QIs achieving attainment over 70% for eligible patients (Table 3). The exceptions to this were QIs assessed in NSTEMI patients; attainment for both QI 2.3 coronary angiography received within 72 hours and QI 4.2 fondaparinux received was just over half of eligible NSTEMI patients.

**Table 3.**
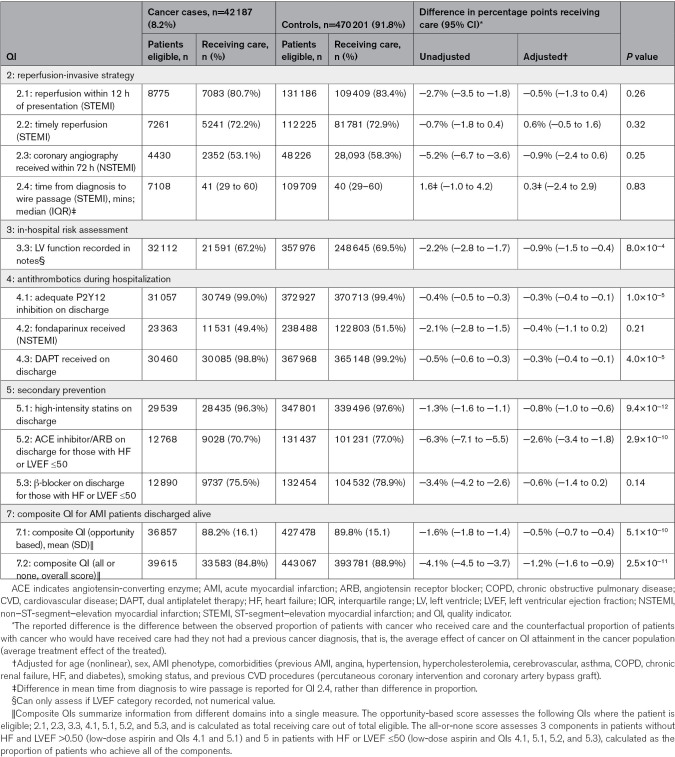
Attainment of QIs for AMI, According to Cancer History

### Reperfusion-Invasive Strategies

**Figure 1.**
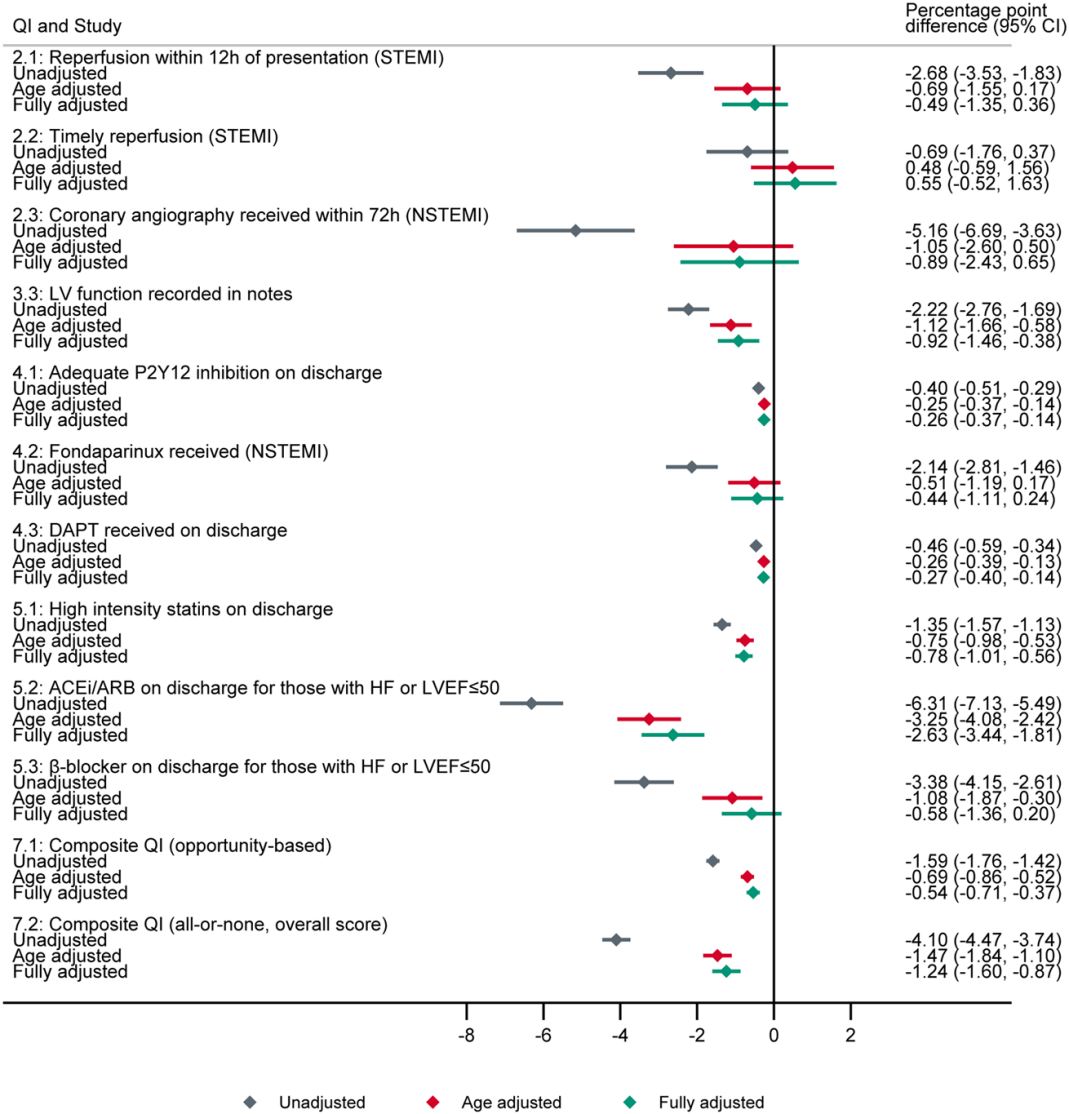
**Difference in attainment of acute myocardial infarction (AMI) quality indicators (QIs) comparing cancer cases to counterfactual controls.** Percentage point difference in QI attainment; unadjusted (gray), adjusted for age (red), and fully adjusted (green). Fully adjusted model adjusted for age (nonlinear), sex, AMI phenotype, comorbidities, smoking status, and previous cardiovascular disease procedures. The error bars represent 95% CIs. QI 2.1: reperfusion within 12 hours of presentation (ST-segment–elevation myocardial infarction [STEMI]). QI 2.2: timely reperfusion (STEMI). QI 2.3: coronary angiography received within 72 hours (non–ST-segment–elevation myocardial infarction [NSTEMI]). QI 3.3: left ventricular (LV) function recorded in notes. QI 4.1: adequate P2Y12 inhibition on discharge. QI 4.2: fondaparinux received (NSTEMI). QI 4.3: dual antiplatelet therapy (DAPT) received on discharge. QI 5.1: high-intensity statins on discharge. QI 5.2: ACE (angiotensin-converting enzyme) inhibitor (ACEi)/angiotensin receptor blocker (ARB) on discharge for those with heart failure (HF) or left ventricular ejection fraction (LVEF) ≤50. QI 5.3: β-blocker on discharge for those with HF or LVEF ≤50. QI 7.1: composite QI (opportunity based). QI 7.2: composite QI (all or none, overall score). QI 2.4: not shown as recorded in minutes (on a continuous scale) and thus is not comparable.

In unadjusted analyses, the percentage of STEMI patients who received reperfusion within 12 hours of presentation (QI 2.1) was 2.7% points lower (95% CI, 1.8–3.5) in patients with cancer compared with controls, while the percentage of NSTEMI patients who received angiography within 72 hours (QI 2.3) was 5.2% points lower (95% CI, 3.6–6.7; Table 3). After adjusting for potential confounders, there was little or no difference of previous cancer on QI attainment for timely reperfusion-invasive strategies (QIs 2.1–2.3; Table 3; Figure 1).

### Pharmacotherapies

There was lower use of antithrombotics (QIs 4.1–4.3) delivered during hospitalization and secondary prevention medications on discharge (QIs 5.1–5.3) in patients with cancer in unadjusted analysis. After adjustment, this small but significant decrease persisted for antiplatelet therapies, ACE (angiotensin-converting enzyme) inhibitors or angiotensin receptor blockers, and statin use on discharge (Table 3; Figure 1).

### Overall Quality of Care

Overall QI attainment was significantly lower in cancer cases compared with controls for many QIs in unadjusted analyses (Table 3), though after adjusting for age, the differences in QI attainment attenuated (Figure 1). Overall, after adjustment, prior cancer was associated with the opportunity-based composite QI in patients with AMI discharged alive (QI 7.1) by half a percentage point (95% CI, 0.4–0.7), while the all-or-nothing composite QI (QI 7.2) was 1.2% points lower (95% CI, 0.9–1.6). Sensitivity analysis showed that clustering by hospital had little effect (Table S7) and results were robust to choice of potential-outcomes analysis method (Figure S2).

### Effect Modification

**Figure 2.**
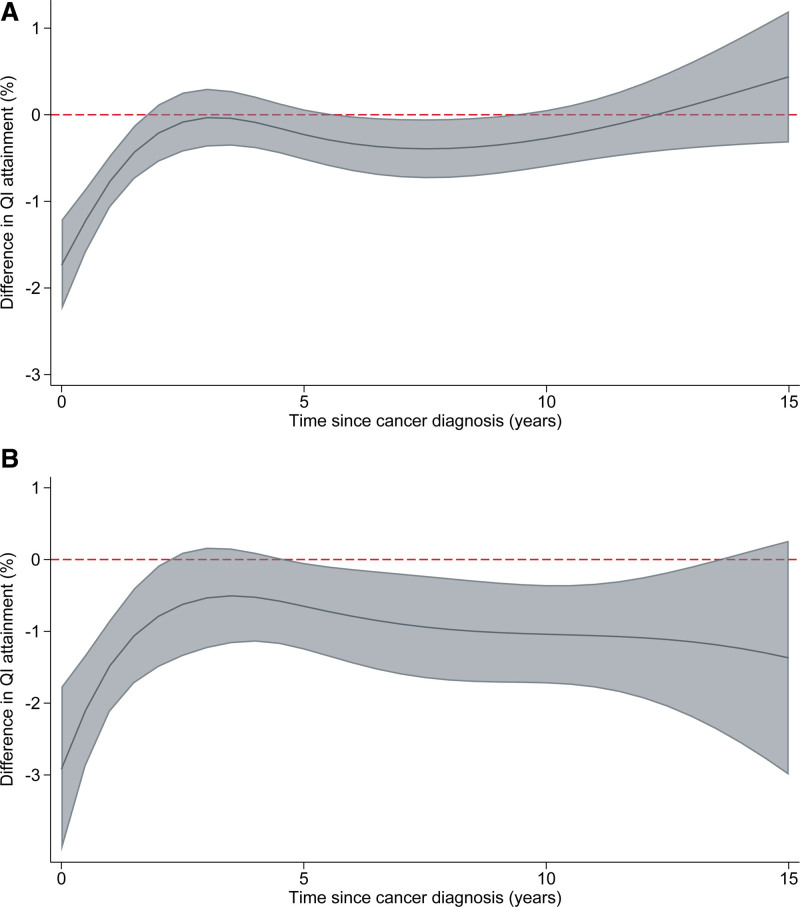
**Difference in composite quality indicator (QI) attainment in cancer cases by time since cancer diagnosis, with counterfactual controls as the reference group. A**, QI 7.1: opportunity-based composite. **B**, QI 7.2: all-or-none composite. Fully adjusted for age (nonlinear), sex, acute myocardial infarction phenotype, comorbidities, smoking status, previous cardiovascular disease procedures, and cancer site. The shaded area represents 95% CIs.

**Figure 3.**
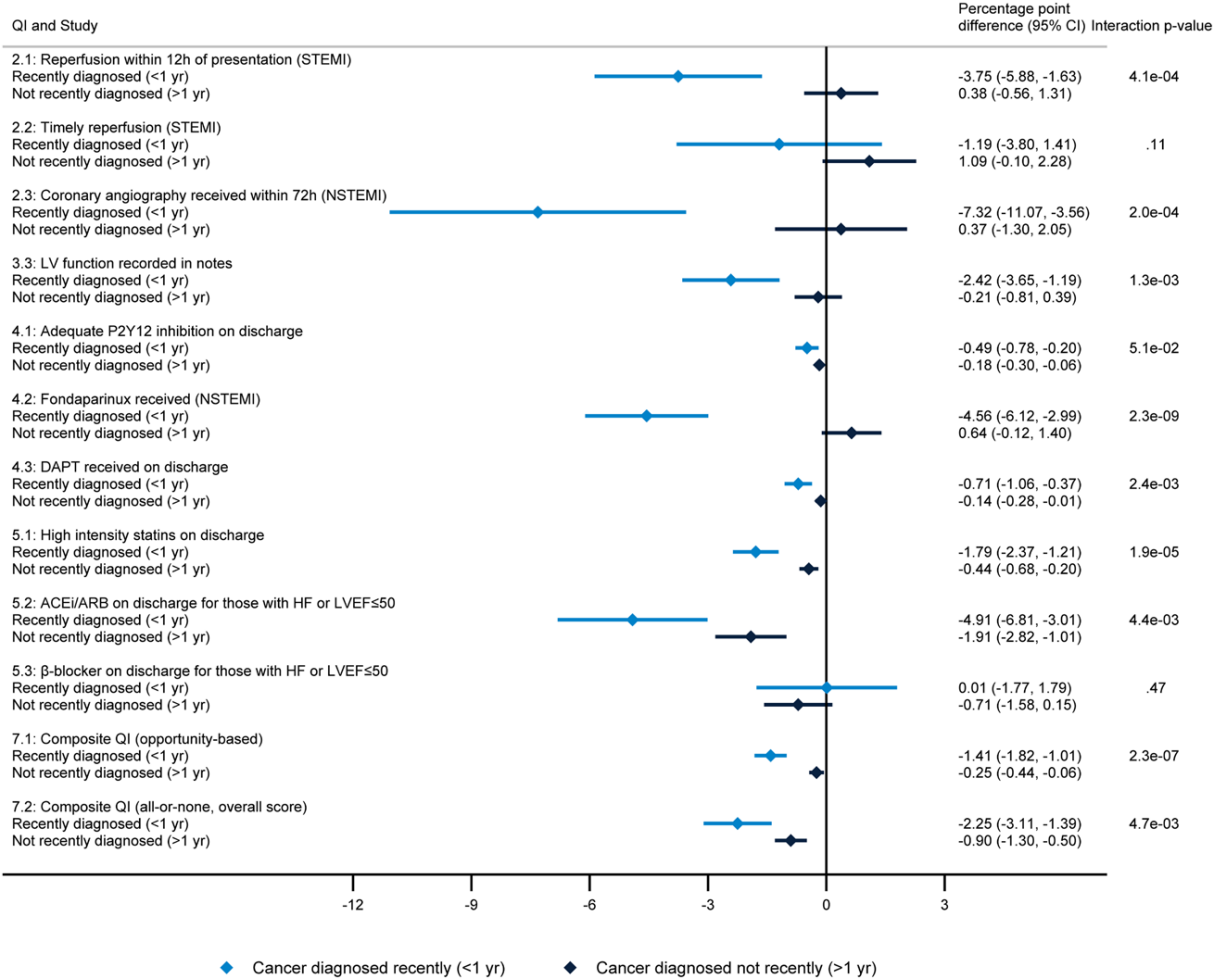
**Difference in quality indicator (QI) attainment for cancers diagnosed recently (<1 year) and cancers diagnosed not recently (>1 year), compared with noncancer controls.** Percentage point difference in QI attainment for cancers diagnosed recently (<1 year before acute myocardial infarction [AMI] hospitalization, light blue) and cancers diagnosed not recently (>1 year before AMI hospitalization, dark blue). Adjusted for age (nonlinear), sex, AMI phenotype, comorbidities (previous AMI, angina, hypertension, hypercholesterolemia, cerebrovascular, asthma, chronic obstructive pulmonary disease, chronic renal failure, heart failure, and diabetes), smoking status, previous cardiovascular disease procedures (percutaneous coronary intervention and coronary artery bypass graft), and cancer site. The error bars represent 95% CIs. ACEi indicates angiotensin-converting enzyme inhibitor; ARB, angiotensin receptor blocker; DAPT, dual antiplatelet therapy; HF, heart failure; LV, left ventricle; LVEF, left ventricular ejection fraction; NSTEMI, non–ST-segment–elevation myocardial infarction; and STEMI, ST-segment–elevation myocardial infarction.

There was evidence of differences in attainment of the composite QIs between cancer cases, with larger differences estimated in patients diagnosed with cancer more recently (Figure 2). Across the QI domains, there was strong evidence of lower attainment for cancers diagnosed within the last year, independent of cancer site (Figure 3). The proportion receiving coronary angiography within 72 hours (QI 2.3) was 7.3% points lower in recently diagnosed cancers compared with controls but was similar in historically diagnosed cancers compared with controls (*P* value for interaction, 2.0×10^−4^). In NSTEMI patients, the proportion receiving fondaparinux was 4.6% points lower in recently diagnosed cancers compared with controls but was similar in historically diagnosed cancers compared with controls (*P* value for interaction, 2.3×10^−9^).

**Figure 4.**
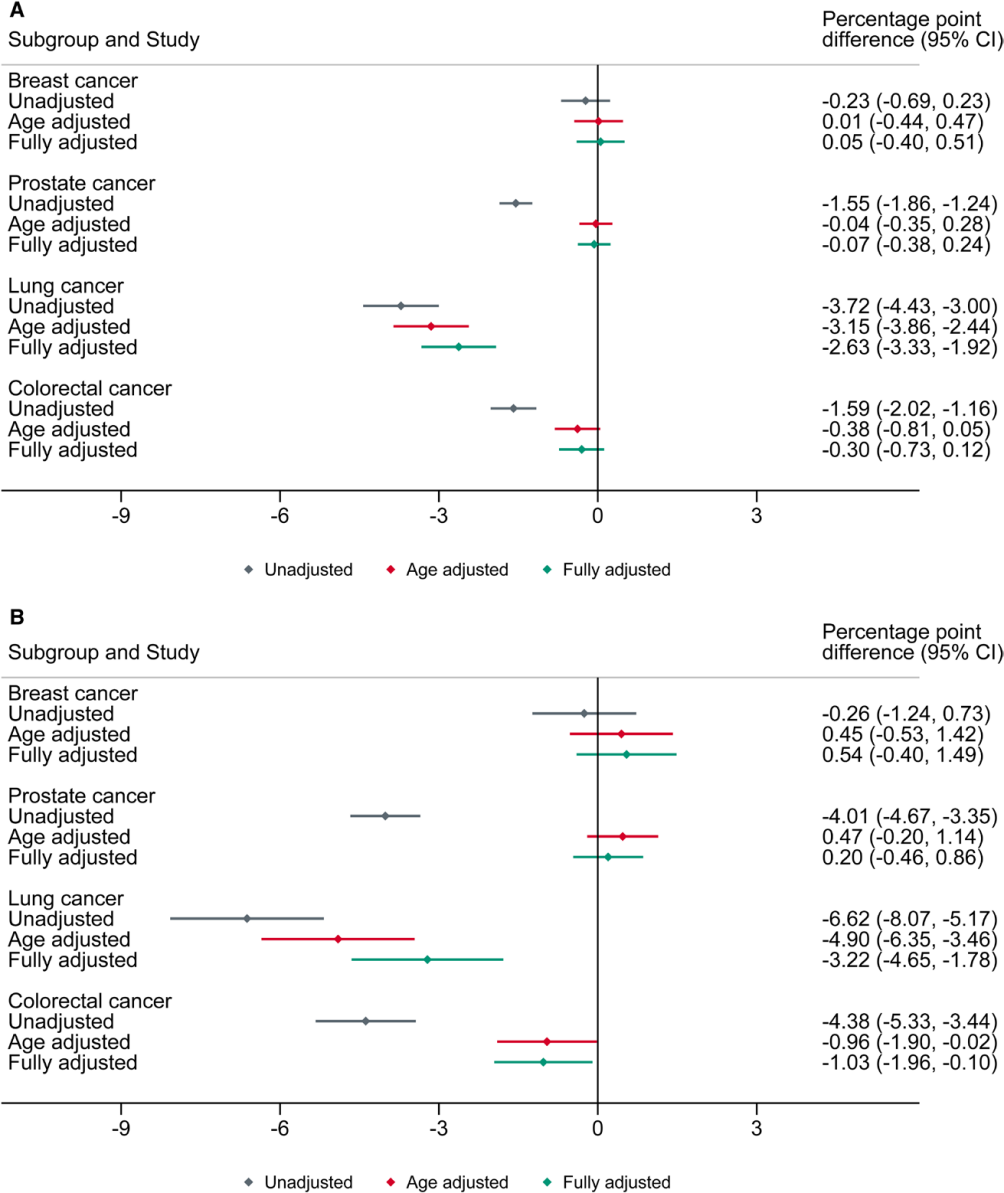
**Difference in attainment of composite acute myocardial infarction quality indicators (QIs) by cancer site, compared with counterfactual controls. A**, QI 7.1: opportunity-based composite. **B**, QI 7.2: all-or-none composite. Percentage point difference in QI attainment; unadjusted (gray), adjusted for age (red), and fully adjusted (green). The error bars represent 95% CIs.

**Figure 5.**
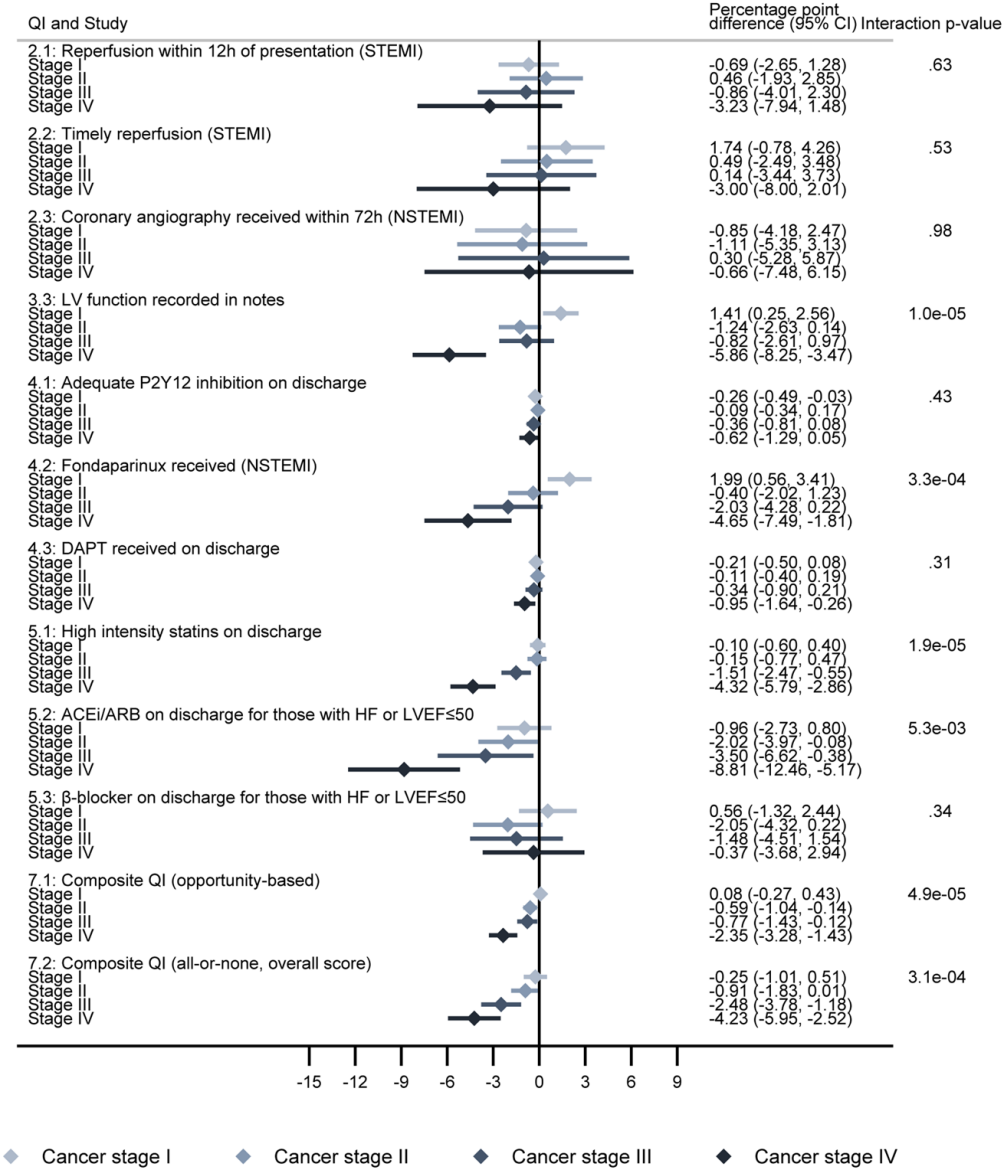
**Difference in quality indicator (QI) attainment by cancer stage, compared with noncancer controls.** The error bars represent 95% CIs. ACEi indicates angiotensin-converting enzyme inhibitor; ARB, angiotensin receptor blocker; DAPT, dual antiplatelet therapy; HF, heart failure; LV, left ventricle; LVEF, left ventricular ejection fraction; NSTEMI, non–ST-segment–elevation myocardial infarction; and STEMI, ST-segment–elevation myocardial infarction.

Analysis by cancer site revealed lower composite QI attainment was predominantly restricted to lung cancer (Figure 4). After adjustment, attainment of QI 7.1 and QI 7.2 was 2.6% points (95% CI, 1.9–3.3) and 3.2% points (95% CI, 1.8–4.6) lower, respectively, in patients with lung cancer compared with noncancer controls, while there was no evidence of a difference in other cancer sites. However, cancer sites other than lung did show poorer QI attainment in those diagnosed in the last year (Figure S3). For patients with lung cancer diagnosed in the last year, QI attainment for composite indicators was up to 6% points lower (Figure S4). There was evidence of an inverse dose-response relationship in QI attainment by cancer stage for many of the domains (Figure 5). Patients with stage III or IV cancer at the time of cancer diagnosis were less likely to receive medications on discharge.

### Survival

Cancer cases had markedly lower all-cause survival following discharge from hospital with AMI even after adjustment for confounders. However, the difference in non–cancer-related deaths was small (Figure S5). Differences were most pronounced in patients with lung cancer (Figure S6). The effect on postdischarge survival of slightly lower composite QI attainment in cancer cases was minimal for most cancer sites (Figure S7). In patients with lung cancer, improving QI attainment to the level observed in noncancer patients was estimated to improve survival by 0.6% (95% CI, 0.4%–0.8%) at 12 months following discharge, translating to 1 death avoided during this period for every 167 additional patients with lung cancer treated with guideline-based care (Figure S7). Maximum QI attainment (if all patients with cancer had achieved the composite QI) was estimated to improve 12-month survival by 1 to 2 percentage points across cancer sites. In non–lung cancer patients, improvements in QI attainment were estimated to improve 12-month postdischarge survival by 0.3% (95% CI, 0.3%–0.4%) in recently diagnosed cancers but with minimal improvement in nonrecent non-lung cancers (Figure S8).

There were large differences in in-hospital mortality between cancer cases and controls (7.9% versus 4.8%; unadjusted difference, 3.0% [95% CI, 2.7–3.3]), which attenuated somewhat after adjustment (difference, 1.4% [95% CI, 1.1–1.7]; Table S8). However, there was little evidence that these differences were mediated by lower QI attainment within patients with cancer overall (Figure S9). The odds ratios of in-hospital mortality associated with improving QI attainment in patients with cancer to that of noncancer controls were small. Further analysis of the impact on in-hospital mortality of improving QI attainment in cancer subgroups was undertaken and showed no clear effect, although CIs were wide due to relatively small numbers of events (data not shown).

## Discussion

We present a national cohort study of the quality of care for patients with AMI and hospitalized in England among 42 187 patients with cancer and 470 201 noncancer controls. We report, first, a lower use of angiography and revascularization in patients with cancer presenting with AMI but no effect of prior cancer on timing of an invasive coronary strategy after adjustment. Second, a lower use of evidence-based antiplatelet medications and secondary prevention medications in the cancer cases. Third, a small overall reduction in measures of quality of AMI care in cancer patients, driven primarily by differences in patients with more recent cancer diagnoses, those with later stage disease, or those with lung cancer. Notably and notwithstanding the differences in care between patients with cancer and their noncancer counterparts, we show that patients with cancer with AMI have worse in-hospital and postdischarge survival but that this is not primarily driven by differences in indicators of AMI care quality.

As cancer treatment advances and cancer-specific survival improves,^2^ an increasing proportion of adverse patient outcomes will be driven by non–cancer-specific causes.^23^ Some of these, including CVD, partly arise as a secondary consequence of cancer and its treatment.^24,25^ Therefore, as the incremental gains in survival from new cancer treatments become smaller, targeting improvements in non–cancer-specific outcomes provides an important opportunity to maintain momentum in continuing to improve patient outcomes. This study confirms previous findings that survival after AMI in patients with cancer is worse than noncancer patients,^26–29^ with >20% mortality at 10 years in patients discharged from hospital. We sought to investigate to what extent this high mortality is due to potentially modifiable differences in quality of care.

Early angiography and revascularization are key tenets of guideline-based management of AMI.^30,31^ Both were used less frequently in patients with cancer with AMI than noncancer patients. There were also fewer patients with cancer achieving the key timeliness metrics for STEMI reperfusion within 30 minutes if treated with fibrinolysis and 60 minutes if treated with primary PCI. Interestingly differences between cancer and noncancer patients reduced when adjusted, predominantly for age. This indicates that the fact that patients with cancer presenting with AMI are older is a critical determinant of their quality of care. Following adjustment, differences in rates of angiography and revascularization persisted, but differences in timeliness metrics were no longer present. This suggests it is the decision to undertake invasive investigation that differs as a consequence of a prior cancer diagnosis, whereas delays are driven primarily by the impact of age. There may also be more subtle procedural differences that have not been investigated in this study. For example, previous studies have shown lower use of drug-eluting stents in patients with cancer^28^ (although these studies pre-date the adoption of drug-eluting stents as the default option used in most PCI procedures). Additional studies with a more in-depth procedural focus will be required to investigate this further.

Key guideline-based medical therapies^30,31^ such as antiplatelet therapies, ACE inhibitors, or angiotensin receptor blockers and use of statins were used less frequently in patients with a prior cancer diagnosis. These findings persisted after adjustment. Understanding the causes of these differences will require further study. While systematic undertreatment of patients with cancer with evidence-based therapies is possible post-AMI, there are several other important potential explanations. The finding that differences are the greatest in patients with a recent cancer diagnosis (<1 year) and lung cancer suggests cancer prognosis has an important impact. Decisions not to treat patients may, of course, be appropriate in the context of palliative management, patient choice, or where there is an increased cancer-related risk of adverse drug effects (such as bleeding with antiplatelet therapies, hypotension, or renal dysfunction with ACE inhibitor/angiotensin receptor blocker).^32^

Predefined QIs provide a means to assess the overall quality of care for AMI.^12^ For this study, the 2017 European Society of Cardiology QIs^11^ were used rather than the recently published 2020 updated QIs^10^ as these best represented contemporaneous practice guidelines to the data set published and were, therefore, readily mapped onto the MINAP data set. Overall, composite assessment demonstrated a small reduction in the combined assessment of QIs in patients with cancer that was attenuated but not abolished by adjustment. Again, age differences between patients with and without previous cancers accounted for a large portion of the observed differences in quality of care; after adjustment for age, these reduced substantially. Interestingly, the differences were more pronounced in patients with a recent cancer diagnosis, those with lung cancer, and late-stage cancers. This heterogeneity strongly suggests that it is less the presence of a prior cancer diagnosis per se, which drives differences in management than the presence of a recent cancer diagnosis, a lung cancer diagnosis, or a cancer with poor prognosis or perceived prognosis. Guidelines for appropriate care and management rarely incorporate recommendations for patients with common comorbidities, despite the prevalence of comorbidities in the CVD population.^32^ The cause of these differences, and whether or not they are appropriate to a cancer population, will require further study.

These findings suggest a prior cancer diagnosis minimally impacts on quality of care for hospitalized AMI in most prevalent cancer patients. A nontargeted approach to improve quality of AMI care in all patients with cancer using these metrics may, therefore, not yield important survival benefits. Further research will be needed to determine whether targeting quality improvements on those with a recent cancer diagnosis, later stage disease, and those with a lung cancer diagnosis could still yield important benefits in outcomes or whether the differences in QIs seen in these groups simply reflect appropriate management of patients with an adverse cancer prognosis. One such improvement could be increasing the communication between oncologists and cardiologists and engaging with cardio-oncology teams when treating this complex patient population.

### Limitations

This is an observational study; we, therefore, cannot conclude that the associations demonstrated are causative. We necessarily used linked English national audit data to ascertain our cancer and AMI cohorts as these data sets provided the level of detail required to assess key quality of case metrics. It is recognized that the MINAP audit ascertains predominantly type 1 AMI cases presenting to cardiology services, and coverage is variable by age and comorbidities. This will not include the population of patients with AMI identified only on hospital episode statistics or primary care records.^33^ The patients with cancer who go on to have AMI who are missing from this analysis will likely be older and with more comorbidities than those included. The results of this analysis are likely conservative estimates of differences, as the missing older frailer patients not treated in specialized clinics are expected to have poorer quality of care and worse survival than those captured. A key advantage of using English data and not having to control for the impact of health insurance on quality of care, due to the NHS System, may limit the generalizability of results to countries without nationalized care. A potential source of bias is the proportion of patients marked in MINAP as not eligible, which occurs more frequently in patients with cancer. It is not possible to determine whether clinicians may determine that a particular intervention is not eligible based primarily on a prior cancer diagnosis in some cases, though it is feasible this may be related, for example, a patient with stage IV cancer on palliative care may not be eligible to undergo invasive treatment strategies. Cause of death information was only available for patients with cancer from linked mortality data; thus for the cause-specific analysis, it was assumed that cancer deaths were negligible in the controls. Furthermore, the underlying cause of death can be unreliable especially for older populations.^34^ Our investigations of the effect of QI attainment on survival post-AMI discharge were conducted separately for each QI and the attainment, or not, of multiple care indicators in combination may have shown a bigger effect on postdischarge outcomes. However, we did assess the composite QIs, which account for attainment across care domains. There is also the potential for unknown biases arising from nonrandomly distributed missing data.

### Conclusions

While the quality of AMI care in patients with cancer is worse than in noncancer patients, this is primarily driven by differences in age and comorbidity between these populations with only small differences attributable to the prior cancer diagnosis per se. Improving quality of care metrics for AMI is likely to have a modest impact on survival of patients with cancer. However, these differences in AMI QIs are exacerbated in patients with prior lung cancer, later stage disease, and a recent cancer diagnosis. It is not yet clear whether these reflect appropriate clinical decision-making or whether targeting quality-of-care improvements in these cancer subpopulations might yield improvements in outcomes after AMI.^32^

## Article Information

### Acknowledgments

We acknowledge the support of the Virtual Cardio-Oncology Research Initiative (VICORI) collaborative and our lead lay representative, Paul Charlton; the National Institute for Cardiovascular Outcomes Research; NHS Digital and their staff, and the National Institute for Cardiovascular Outcomes Research audit leads. Particular thanks to David Forman as external member for chairing the VICORI project review panel. This study was supported by the National Institute for Health and Care Research (NIHR) Applied Research Collaboration East Midlands and Leicester NIHR Biomedical Research Centre. The views expressed are those of the authors and not necessarily those of the NIHR or the Department of Health and Social Care. National Heart Failure Audit is commissioned, as part of the “National Cardiac Audit Programme” by the Healthcare Quality Improvement Partnership. This research used the Advanced Leicester Information and Computational Environment High Performance Computing Facility at the University of Leicester. This work uses data that have been provided by patients and collected by the National Health Service as part of their care and support. The data are collated, maintained, and quality assured by the National Cancer Registration and Analysis Service, which is part of NHS Digital. Conceptualization: Dr Teece, Dr Sweeting, Dr Hall, M.D. Peake, Dr Gale, and D. Adlam; data curation: Dr Teece, Dr Sweeting, and Dr Hall; formal analysis: Dr Teece and Dr Sweeting; funding acquisition: D. Adlam, M.D. Peake, J. Deanfield, Dr de Belder, Dr Rutherford, and Dr Lambert; investigation: Dr de Belder, J. Deanfield, C. Weston, L. Paley, M.D. Peake, Dr Gale, and D. Adlam; methodology: Dr Teece, Dr Sweeting, Dr Rutherford, Dr Lambert, and Dr Gale; project administration: Dr Teece and Dr Sweeting; software: Dr Rutherford, and Dr Lambert; visualization: Dr Teece and Dr Sweeting; writing–original draft: Dr Teece, Dr Sweeting, and D. Adlam; writing–review and editing: Dr Teece, Dr Sweeting, Dr Hall, B. Coles, Dr Oliver-Williams, C. Welch, Dr de Belder, J. Deanfield, C. Weston, Dr Rutherford, L. Paley, Dr Kadam, Dr Lambert, M.D. Peake, Dr Gale, and D. Adlam. Dr Sweeting and D. Adlam are the guarantors.

### Sources of Funding

This study was jointly funded by Cancer Research UK (C53325/A21134) and the British Heart Foundation (SP/16/5/32415). The funders had no role in the study design; in the collection, analysis, or interpretation of the data; in the writing of the report; or in the decision to submit the manuscript for publication.

### Disclosures

Dr Teece, Dr Sweeting, B. Coles, Dr Oliver-Williams, Dr Welch, Dr Rutherford, Dr Lambert, D. Adlam, and M.D. Peake had financial support from the British Heart Foundation and Cancer Research UK for the submitted work. D. Adlam has received research funding and in kind support for unrelated research from AstraZeneca, Inc. He has received an educational grant from Abbott Vascular, Inc, to support a clinical research fellow for unrelated research. He has also conducted consultancy for General Electric Inc., to support research funds for unrelated research. B. Coles previously received funding from Novo Nordisk. J. Deanfield had financial support from the British Heart Foundation in the previous 3 years. Dr Sweeting is a full-time employee of AstraZeneca. C. Weston is the clinical lead of the Myocardial Ischaemia National Audit Project. Dr de Belder reports data safety and monitoring board membership of the UK GRIS (UK GRACE Risk Score Intervention Study) trial, is chair of the ARREST (a randomized trial of expedited transfer to a cardiac arrest center for non-ST elevation out-of-hospital cardiac arrest) trial Steering Committee, and is an executive member of the DAPA-MI (dapagliflozin effects on cardiovascular events in patients with an acute heart attack) trial. The other authors report no conflicts.

### Supplemental Material

Supplemental Methods

Figures S1–S9

Tables S1–S8

References 35–39

RECORD Reporting Checklist

## Supplementary Material


