# Supplementary material for: Impact of a Prior Cancer Diagnosis on Quality of Care and Survival Following Acute Myocardial Infarction: Retrospective Population-Based Cohort Study in England
Source: Circ Cardiovasc Qual Outcomes. 2023 Jun 20;16(6):e009236. doi: 10.1161/CIRCOUTCOMES.122.009236 (PMC10281182; doi:10.1161/CIRCOUTCOMES.122.009236)
Supplement: Supplementary file 1 [file hcq-16-e009236-s001.pdf]

Supplemental Material

For "The impact of prior cancer diagnosis on quality of care and survival after acute myocardial infarction: retrospective population-based cohort study"

Lucy Teece[*], Michael J Sweeting[*], Marlous Hall, Briana Coles, Clare Oliver-Williams, Cathy A. Welch, Mark A. de Belder, John Deanfield, Clive Weston, Mark J Rutherford, Lizz Paley, Umesh T. Kadam, Paul C Lambert, Michael D. Peake[ǂ], Chris P Gale[ǂ], and David Adlam[ǂ] on behalf of the VICORI collaborative

Contents

Supplementary Methods 3

European Society of Cardiology Quality Indicators 3

Estimating differences in QI attainment using a potential outcomes framework 3

Estimates of effect modification 4

Post-discharge survival analysis 5

Effect of suboptimal care on post-discharge survival 6

Missing data analysis 6

Figure S1. Criteria for identifying ST-segment elevation and non-ST-segment elevation myocardial infarction hospitalisations using Myocardial Ischaemia National Audit Project (MINAP) data 8

Figure S2. Comparison of different modelling techniques for assessing differences in quality indicator attainment 9

Figure S3. Difference in quality indicator attainment for lung cancers, other site cancers diagnosed recently (< 1 year) and other site cancers diagnosed not recently (>1 year), compared to non-cancer controls 10

Figure S4. Difference in composite quality indicator attainment in lung cancer cases by time since cancer diagnosis, with non-cancer controls as the reference group 12

Figure S5. All cause and non-cancer related survival for cancer cases, controls and counterfactual controls (adjusted) 13

Figure S6. Non-cancer related net survival by cancer type compared to controls and counterfactual controls (adjusted) 14

Figure S7. Impact of improved composite quality indicator attainment (7.2) on differences in all-cause survival compared to current quality indicator attainment, by cancer type 15

Figure S8. Impact of improved composite quality indicator attainment (7.2) on differences in all-cause survival compared to current quality indicator attainment, by recent and non-recent non-lung cancer 16

Figure S9. Impact on in-hospital mortality of improved versus current quality indicator attainment in cancer patients 17

Table S1. Summary of quality indicators (QIs) assessed 18

Table S2. Tumour characteristics of patients with a cancer diagnosis in the 15 years preceding AMI hospitalisation (most recent diagnosis). 19

Table S3. Missing data, n (%), in baseline characteristics of patients hospitalised with acute myocardial infarction, according to cancer history 20

Table S4. Baseline characteristics of patients hospitalised with acute myocardial infarction for cancer cases versus age-matched controls........ 21

Table S5. Early management and in-hospital treatment of patients admitted for AMI for cancer cases versus age-matched controls 23

Table S6. Eligible for quality indicator assessment 24

Table S7. Comparison of different standard error selection when assessing differences in quality indicator attainment 25

Table S8. In-hospital mortality following AMI admission in cancer cases, controls and counterfactual (adjusted) controls 27

The RECORD statement – checklist of items, extended from the STROBE statement, that should be reported in observational studies using routinely collected health data. 28

Supplementary Methods

European Society of Cardiology Quality Indicators

Features of hospital treatment during admission for AMI were taken from the European Society of Cardiology Acute Cardiovascular Care Association quality indicators (QIs) for the evaluation of care[11]. The original guidelines contain twenty QIs (twelve main and eight secondary) across seven domains related to (1) centre organization; (2) reperfusion or invasive strategy; (3) in-hospital risk assessment; (4) antithrombotic treatment during hospitalisation; (5) secondary prevention discharge treatments; (6) patient satisfaction, and (7) composite QIs that compound information across the other domains. MINAP was used to assess attainment for thirteen of the QIs based on previous research that has mapped components of the QIs to relevant MINAP data items[12,14], listed in Supplementary Table S2. The remaining QI's either could not be assessed using the available data, including documentation of GRACE[1] and CRUSADE[35] risk scores in medical notes, patient satisfaction, and mortality rate adjusted for GRACE score, or reported centre organisation QIs, which achieved 100% attainment.

Estimating differences in QI attainment using a potential outcomes framework

We utilised the potential outcomes framework specifically, via standardisation methods[36], to estimate the attainment of the QIs in those with cancer, and understand how this would have differed had the same population not had cancer diagnoses in the previous 15 years. In order to assess differences in attainment of each AMI QI between cancer cases and controls, a series of multivariable regression models with confounder adjustment for each QI were fitted with robust standard errors. Logistic regression models were used for all QIs measured on a binary scale, except for QIs 2·4 and 7·1 which were continuous measures and therefore assessed using linear regression. QI attainment was initially adjusted for the non-linear

effects of age at AMI admission using restricted cubic splines estimated with four degrees of freedom with knots placed at equally spaced centiles of the distribution. A second, fully adjusted model additionally included sex, AMI phenotype (STEMI/NSTEMI), comorbidities (previous AMI, angina, hypertension, hypercholesterolaemia, cerebrovascular disease, asthma, chronic obstructive pulmonary disease, chronic renal failure, heart failure, diabetes), smoking status, and previous cardiovascular disease procedures (PCI, coronary artery bypass graft (CABG)). Using standardisation, we calculated the average difference in QI attainment due to cancer, standardizing over patient characteristics of the cancer cases (i.e. calculating the average exposure effect in the exposed group. We used the *teffects ra* command in Stata[37] to calculate the average difference in QI attainment due to cancer, standardising over patient characteristics of the cancer cases (i.e. calculating the average exposure effect in the exposed group). This provides a marginal estimate of the average treatment effect in the treated (ATET). Effect modification was investigated with differences in QI attainment estimated by time since cancer diagnosis, and by cancer stage. These analyses were additionally adjusted for cancer site due to confounding between site and time since diagnosis and stage. Further analyses were conducted stratified by four common tumour sites: breast, prostate, lung, and colorectal. We conducted a sensitivity analysis to determine whether heteroskedascity across hospital sites altered QI attainment results. Patients were clustered by hospital, results obtained with cluster robust standard errors were compared to those with robust standard errors. To assess the robustness of the average treatment effect estimates obtained from the multivariable regression approach, we conducted a sensitivity analysis using three alternative potential-outcomes approaches for the composite QIs including inverse-probability weighting, augmented inverse-probability weighting, and inverse-probability weighting regression adjustment.

Estimates of effect modification

Effect modification by time since cancer diagnosis was investigated by incorporating time since diagnosis into the models as a continuous measure using restricted cubic splines with 4 degrees of freedom to allow for non-linearity. We allowed an interaction between each of the confounders and each of the basis functions of the cubic spline. We additionally adjusted for the main effects of cancer site. A similar approach was taken to investigate effect modification by cancer stage, with stage entered as a four level categorical variable. Stratified analyses were then conducted within four common tumour sites: (1) breast (women only), (2) prostate (men only), (3) lung, and (4) colorectal. Control groups for breast and prostate cancer were restricted to women and men, respectively. A further analysis investigating effect modification by time since diagnosis stratified by tumour site was conducted in those sites with a significant modifying effect.

Post-discharge survival analysis

Flexible parametric survival models were used to examine survival up to one year post-hospital discharge[21]. A restricted cubic spline was used to model the baseline cumulative hazard of mortality, with four degrees of freedom (selected using AIC criteria from models with 2-6 degrees of freedom). Cancer was interacted with analysis time using a further restricted cubic spline to allow a non-proportional effect of cancer over follow-up. Adjusted analyses included age, sex, and AMI phenotype as covariates, with a non-linear effect of age modelled using a restricted cubic spline. Two way interactions between cancer and age, sex and AMI phenotype were included. Standardised post-discharge curves were obtained for cancer and counterfactual controls, standardising across the covariate distribution of cancer patients. Non-cancer related cause-specific mortality was investigated assuming no cancer deaths in the control group and using underlying cause of death information in cancer cases (censoring deaths from cancer; ICD-10 Chapter II, Neoplasms (C00-C96)) in order to obtain net survival estimates.

Effect of suboptimal care on post-discharge survival

The effect of suboptimal quality indicator attainment on survival following AMI-discharge was investigated in cancer patients. A mediation approach was taken with a Royston-Parmar multivariable flexible parametric survival model fitted to the outcome (post-discharge survival) for cancer cases only, adjusting for the quality indicator under investigation, age, sex, and AMI phenotype, and time since cancer. Continuous variables (age and time since cancer) were modelled using restricted cubic splines to allow for non-linearity. A logistic regression model was fitted to the mediator (quality indicator). The natural indirect effect of cancer on survival that acts through suboptimal QI attainment was estimated using standardisation, weighted by the mediation distribution in cancer cases and controls. This provides an estimate of survival in a cancer population had they received the same level of AMI care as an equivalent non-cancer population. The analyses were then repeated with in-hospital mortality as the outcome, modelled using logistic regression. Further details of this approach are described in Syriopoulou[38]. Only attainment of pre-discharge QIs were used in the analysis of in-hospital mortality (QI domains 2, 3 and 4.2).

Missing data analysis

Missing data was accounted for using multiple imputation with chained equations. A separate imputation model was used for each QI outcome since the number of individuals contributing to each outcome differed (Table 3 in manuscript). The imputation models included the following covariates: QI attainment, case/control status, smoking status, diabetes, comorbidities, previous CVD procedures, previous CVD medications, cancer diagnosis date, time since cancer, cancer stage, cancer site, age, sex, AMI phenotype. Conditional imputation was used to impute cancer-specific variables only for cancer cases. Thirty imputed datasets were generated per QI. Regression models were fitted to each imputed dataset with estimates

pooled using Rubin’s rules. It was decided not to impute cancer stage as a large number of records were missing stage information (mainly reflecting lack of recording of cancer stage in earlier registration years; 15% missing in stage 2015 rising to >80% missing stage in diagnosis years before 2005). Therefore effect modification analyses by cancer stage were performed on complete cases only.

Figure S1. Criteria for identifying ST-segment elevation and non-ST-segment elevation myocardial infarction hospitalisations using Myocardial Ischaemia National Audit Project (MINAP) data

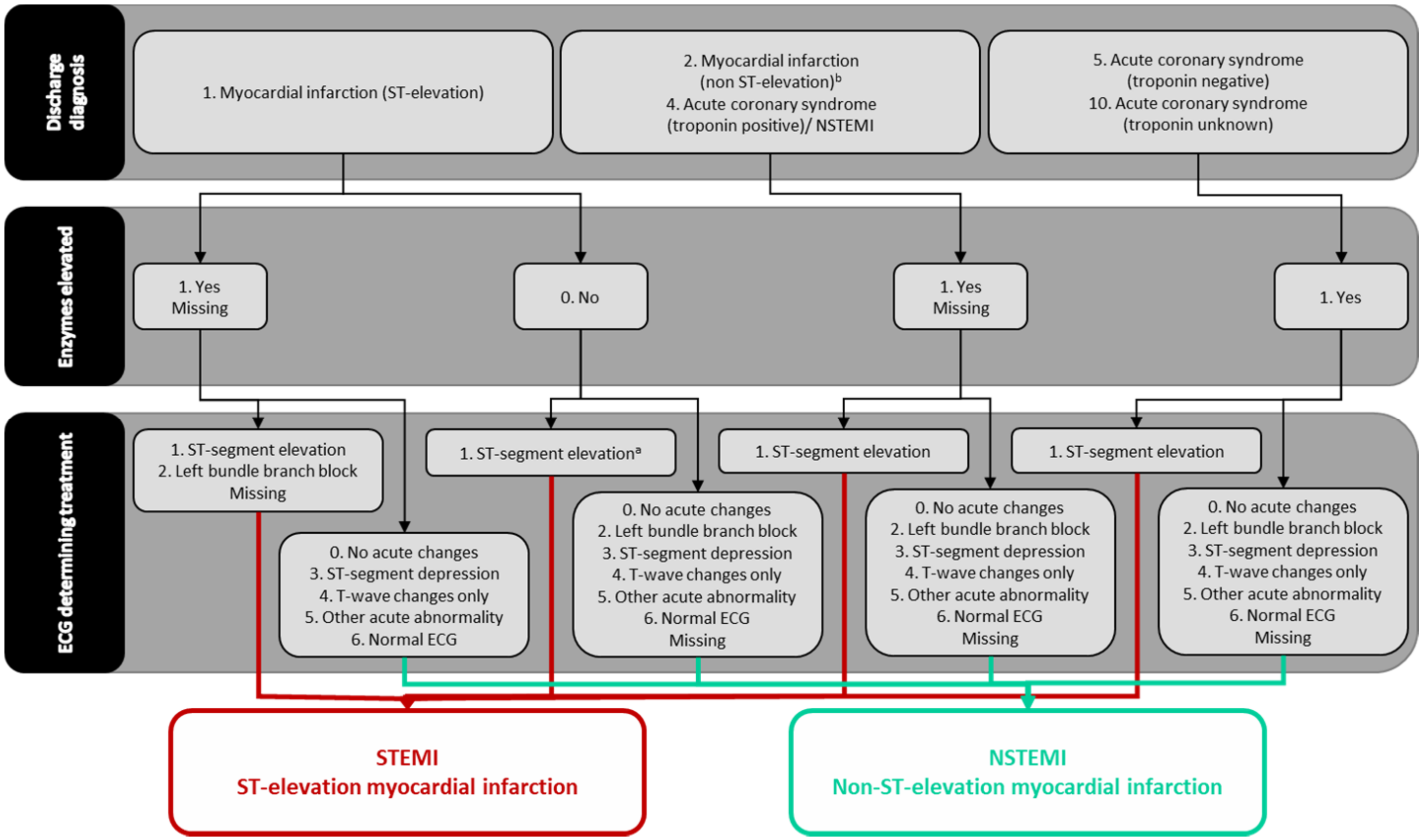

Modified from criteria used in the Cardiovascular disease research using linked bespoke studies and electronic health records (CALIBER) programme[39]

[a]This combination is coded as “Not Acute MI” in CALIBER.
[b]This discharge diagnosis code was not present in MINAP for the CALIBER study.
Abbreviations: AMI, acute myocardial infarction; ECG, electrocardiogram; STEMI, ST-elevation myocardial infarction; NSTEMI, non-ST-segment elevation myocardial infarction.

Figure S2. Comparison of different modelling techniques for assessing differences in quality indicator attainment

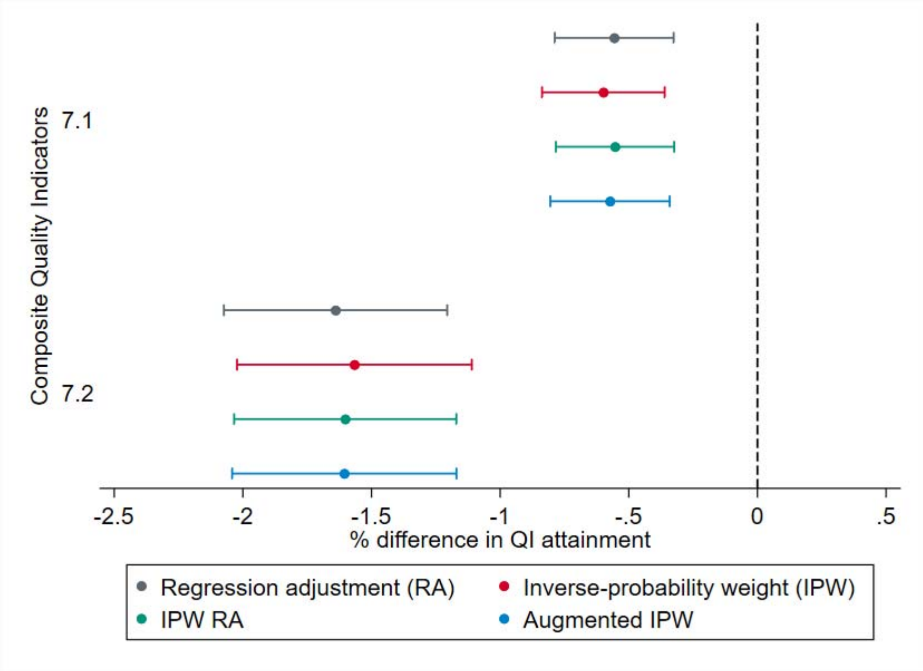

Difference in attainment calculated for composite QIs using various potential-outcomes approaches, including regression adjustment (RA, inverse-probability weighting (IPW), IPW regression adjustment (IPWRA), and augmented IPW (AIPW), adjusted for age (non-linear), sex, AMI phenotype, comorbidities, smoking status, and previous CVD procedures. The error bars represent 95% confidence intervals.

Figure S3. Difference in quality indicator attainment for lung cancers, other site cancers diagnosed recently (< 1 year) and other site cancers diagnosed not recently (>1 year), compared to non-cancer controls

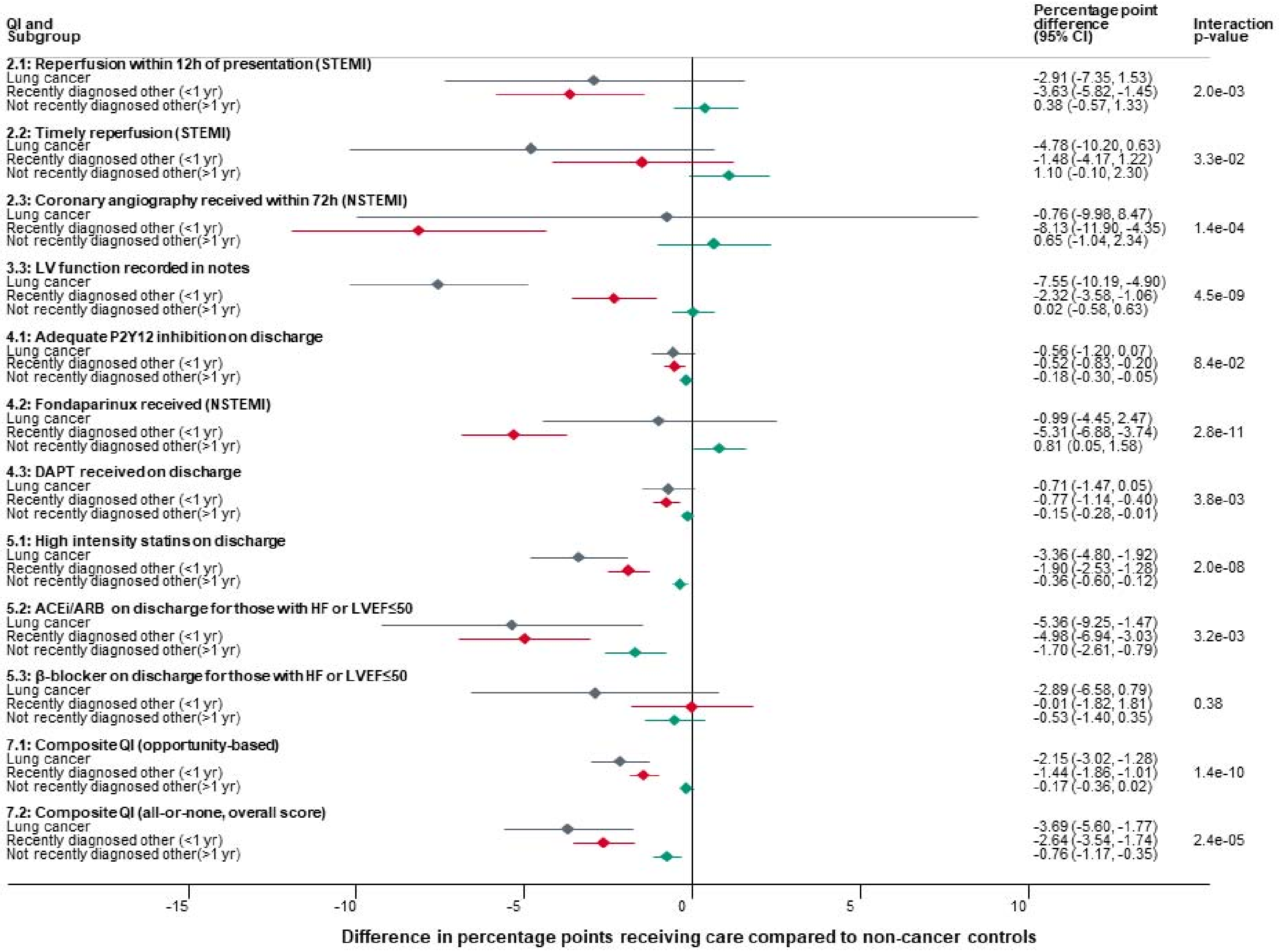

The error bars represent 95% confidence intervals.

Abbreviations: QI, quality indicator; CI, confidence interval; STEMI, ST-segment elevation myocardial infarction; NSTEMI, non-ST elevation myocardial infarction; LV, left ventricular; DAPT, dual antiplatelet therapy; ACEi, angiotensin-converting enzyme inhibitors; ARB, angiotensin receptor blockers; HF, heart failure; LVEF, left ventricular ejection fraction.

Figure S4. Difference in composite quality indicator attainment in lung cancer cases by time since cancer diagnosis, with non-cancer controls as the reference group

a) Quality indicator 7.1: Opportunity based composite

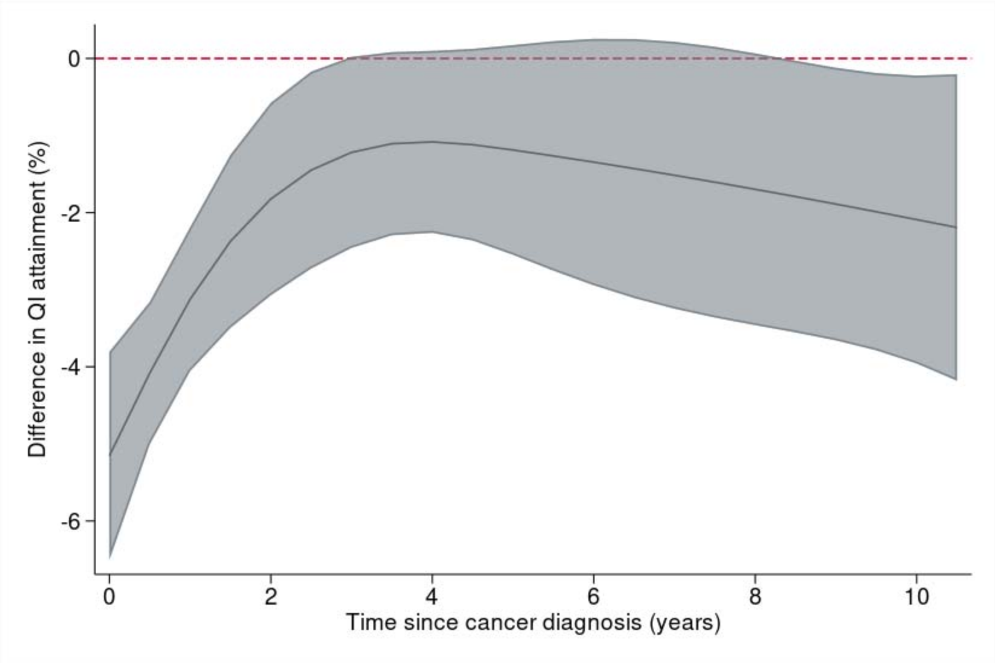

b) Quality indicator 7.2: All-or-none composite

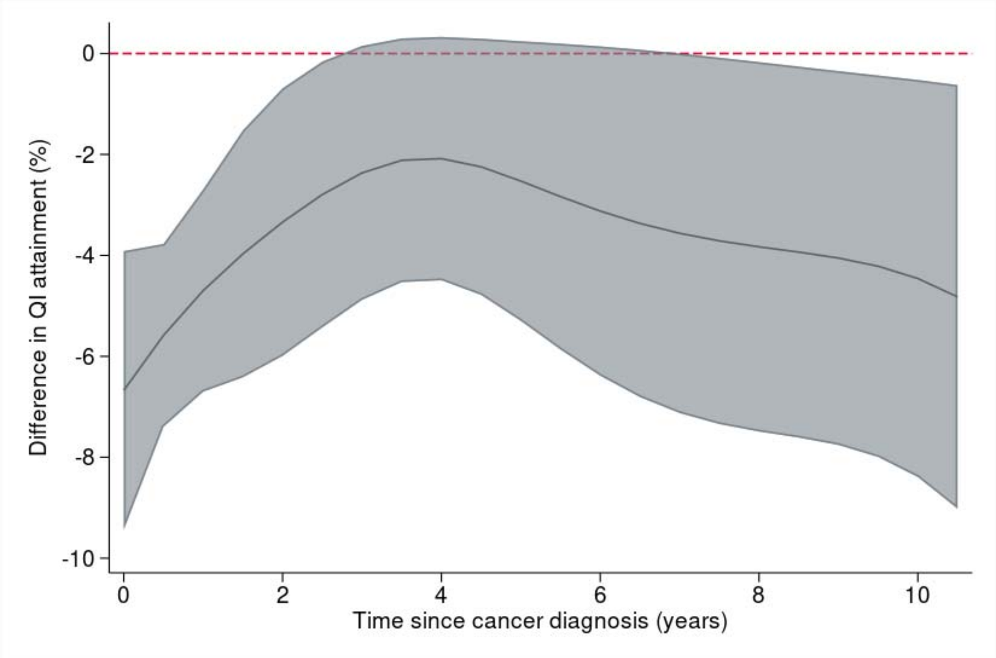

The shaded area represents 95% confidence intervals.
Abbreviations: QI, quality indicator.

Figure S5. All cause and non-cancer related survival for cancer cases, controls and counterfactual controls (adjusted)

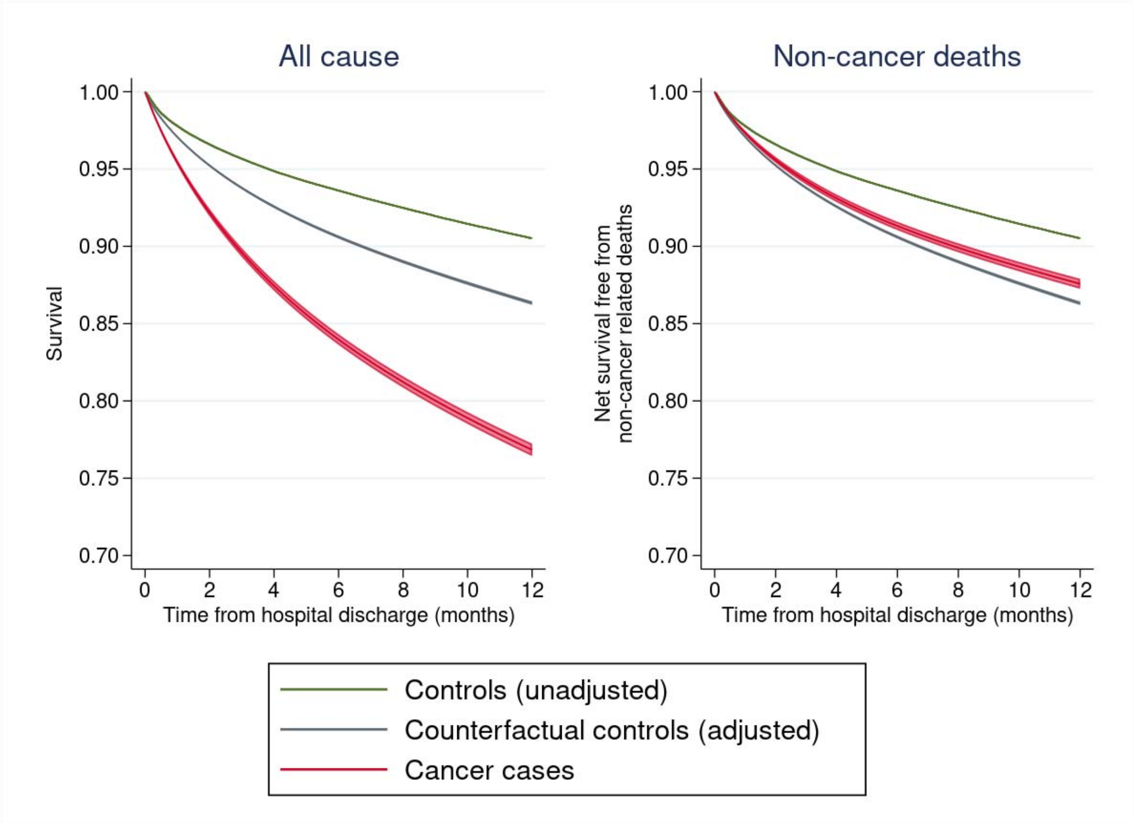

Counterfactual controls (adjusted) shows the survival in controls standardised to the baseline characteristics of the cancer population. The shaded area represents 95% confidence intervals.

Figure S6. Non-cancer related net survival by cancer type compared to controls and counterfactual controls (adjusted)

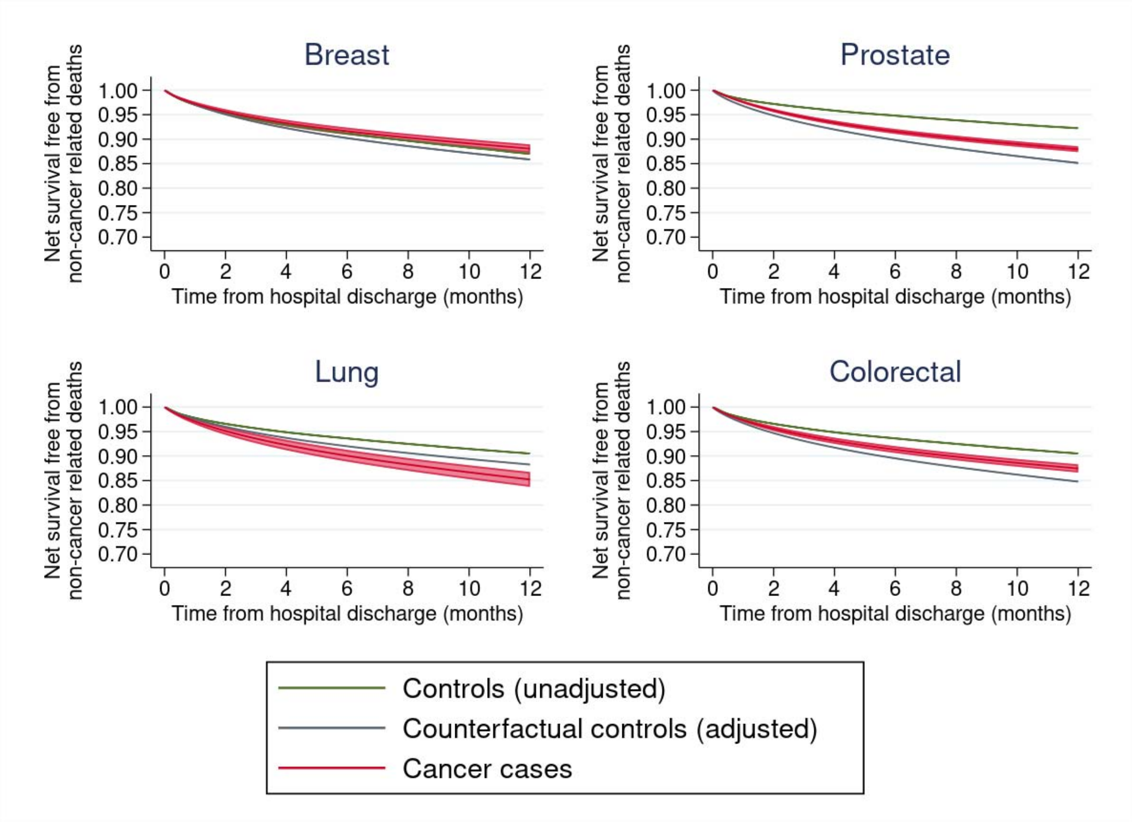

Counterfactual controls (adjusted) shows the survival in controls standardised to the baseline characteristics of the cancer population. The shaded area represents 95% confidence intervals.

Figure S7. Impact of improved composite quality indicator attainment (7.2) on differences in all-cause survival compared to current quality indicator attainment, by cancer type

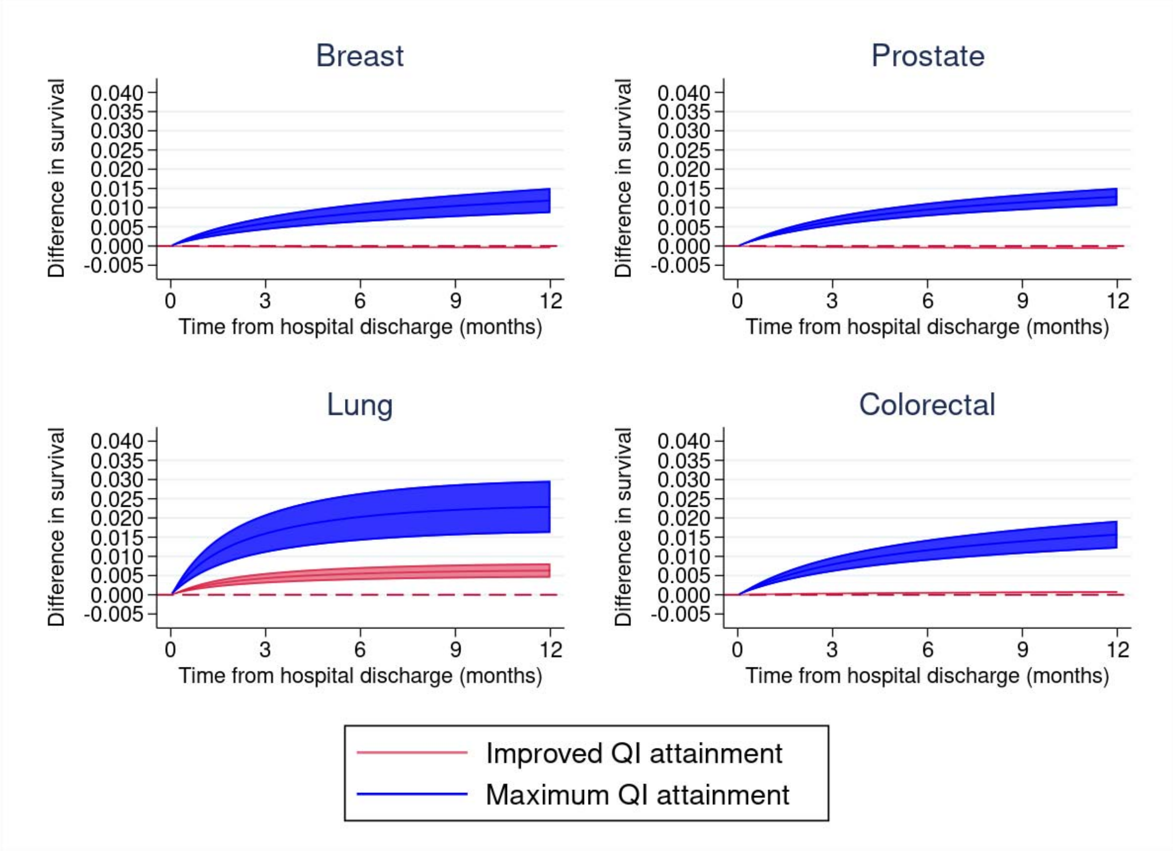

Improved QI attainment: Survival in cancer patients if there was an increase in quality indicator attainment to levels seen in non-cancer patients.

Maximum QI attainment: Survival in cancer patients if all patients attained maximum level of care as defined by the composite quality indicator.

The shaded area represents 95% confidence intervals.

Figure S8. Impact of improved composite quality indicator attainment (7.2) on differences in all-cause survival compared to current quality indicator attainment, by recent and non-recent non-lung cancer

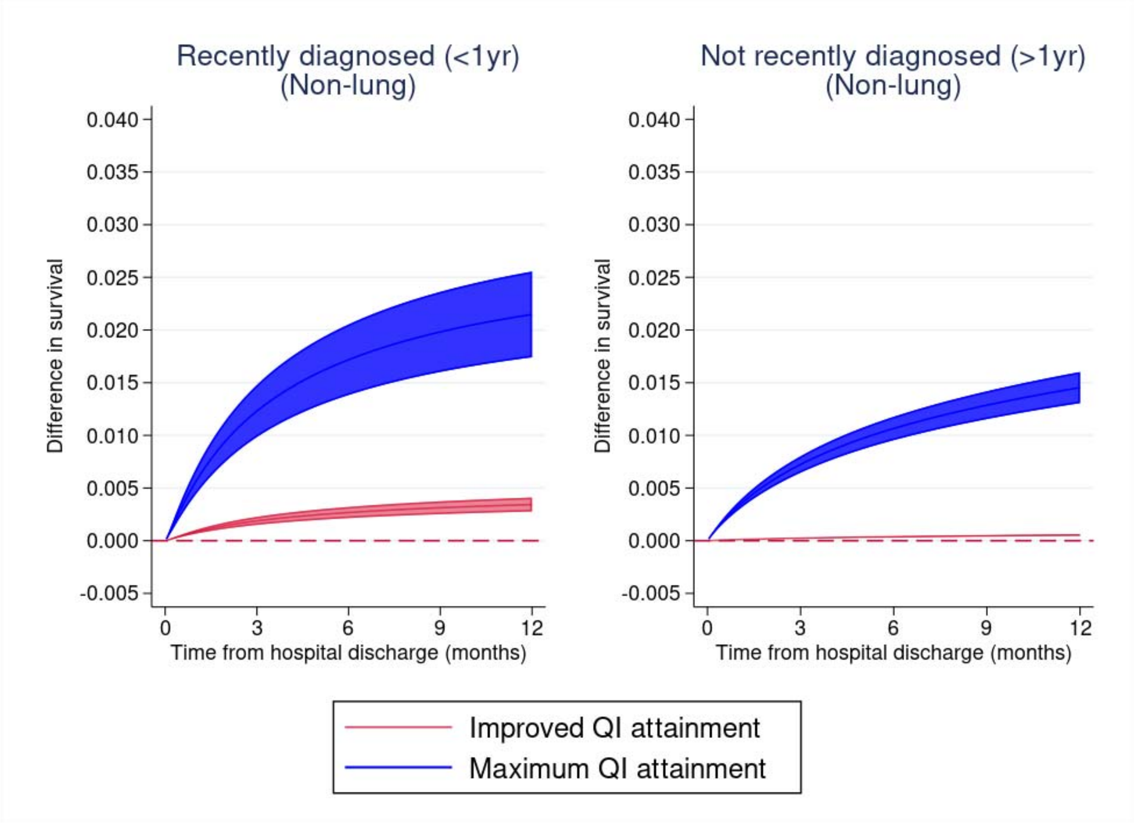

Improved QI attainment: Survival in cancer patients if there was an increase in quality indicator attainment to levels seen in non-cancer patients.

Maximum QI attainment: Survival in cancer patients if all patients attained maximum level of care as defined by the composite quality indicator.

The shaded area represents 95% confidence intervals.

Figure S9. Impact on in-hospital mortality of improved versus current quality indicator attainment in cancer patients

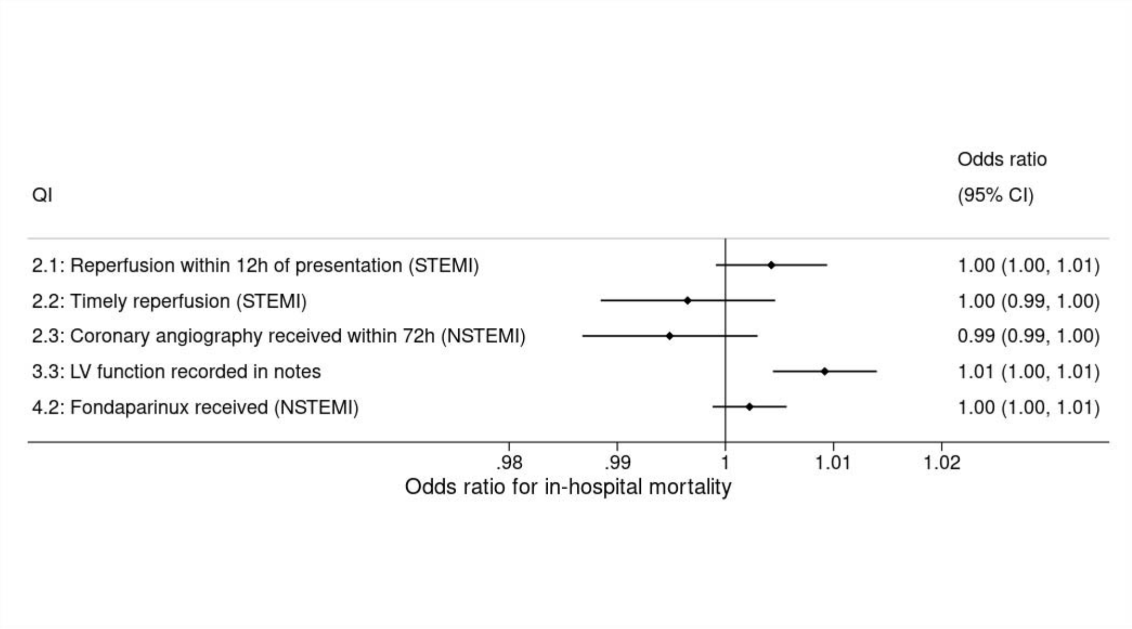

The shaded area represents 95% confidence intervals.
Abbreviations: STEMI, ST-segment elevation myocardial infarction; NSTEMI, non-ST elevation myocardial infarction; LV, left ventricular.

Table S1. Summary of quality indicators (QIs) assessed

Quality Indicator	Eligibility
**2: Reperfusion/invasive strategy**	
**2.1:** Reperfusion within 12h of presentation (STEMI)	All STEMI patients eligible for reperfusion (onset of symptoms to diagnosis <12 h, without contraindication or patient refusal)
**2.2:** Timely reperfusion (STEMI)	All STEMI patients eligible for reperfusion by primary PCI (onset of symptoms to diagnosis <12 h, without contraindication or patient refusal).
**2.3:** Coronary angiography received within 72h (NSTEMI)	All NSTEMI patients at high-intermediate ischaemic risk without contraindications or patient refusal
**2.4:** Time from diagnosis to wire passage (STEMI): mins, med [IQR]	All STEMI patients who receive reperfusion
**3: In Hospital risk assessment**	
**3.3:** LV function recorded in notes	All AMI patients
**4: Anti-thrombotics during hospitalisation**	
**4.1:** Adequate $P2Y_{12}$ inhibition on discharge	STEMI and NSTEMI patients alive at discharge and without contraindications to P2Y12 inhibitors
**4.2:** Fondaparinux received (NSTEMI)	All NSTEMI patients with eGFR ⩾ 20 ml/min, not candidates for urgent invasive strategy.
**4.3:** DAPT received on discharge	All STEMI and NSTEMI patients, without contra indications to dual antiplatelet therapy
**5: Secondary prevention**	
**5.1:** High intensity statins on discharge	STEMI and NSTEMI patients alive at discharge and without contraindications, refusal, side effects, allergy, or history of intolerance to high-intensity statin therapy
**5.2:** ACEi/ARB on discharge for those with HF or LVEF≤50	All AMI patients who have heart failure or a LVEF⩽0.40, and who are eligible for ACEI/ARBs (no hypotension, acute renal failure, hyperkalaemia, contraindications, refusal, side effects or allergy).
**5.3:** β-blocker on discharge for those with HF or LVEF≤50	All AMI patients who have heart failure or a LVEF⩽0.40, and are eligible for beta-blockers (no evidence of a low output state, increased risk for cardiogenic shock, PR interval >0.24 s, second- or third-degree heart block, active asthma, or reactive airways disease).
**7: Composite QI for AMI patients discharged alive**	
**7.1:** Composite QI (opportunity-based): Mean (SD)	All AMI patients discharged: sum of points (one point for each applicable indicator, according to patient and centre characteristics).
**7.2:** Composite QI (all-or-none, overall score)	Calculated on 3 individual QIs in patients without heart failure and with LVEF>0.40.<br>• Low-dose aspirin.<br>• P2Y12 inhibitor (unless documented contraindication).<br>• High-intensity statins.<br>Calculated on 5 individual QIs in patients with heart failure or with LVEF⩽0.40.<br>• Low-dose aspirin.<br>• P2Y12 inhibitor (unless documented contraindication).<br>• High-intensity statins.<br>• ACEI (or ARB if intolerant to ACEI) in patients with clinical evidence of heart failure or LVEF⩽0.40.<br>• Beta-blockers (unless clear contraindication) in patients with clinical evidence of heart failure or LVEF⩽0.40.

Abbreviations QI, quality indicator; STEMI, ST-segment elevation myocardial infarction; PCI, percutaneous coronary intervention; NSTEMI, non-ST elevation myocardial infarction; IQR, interquartile range; LV, left ventricular; eGFR, estimated glomerular filtration rate; DAPT, dual antiplatelet therapy; ACEi, angiotensin-

converting enzyme inhibitors; ARB, angiotensin receptor blockers; HF, heart failure; LVEF, left ventricular ejection fraction; AMI, acute myocardial infarction; SD, standard deviation, AMI, acute myocardial infarction.

Table S2. Tumour characteristics of patients with a cancer diagnosis in the 15 years preceding AMI hospitalisation (most recent diagnosis).

	Bladder N=2,283	Breast N=5,236	Colorectal N=6,043	Kidney N=1,757	Lung N=2,870	Prostate N=11,498	Other N=12,500
**Stage at diagnosis**							
I	442 (19.4%)	1,393 (26.6%)	521 (8.6%)	335 (19.1%)	621 (21.6%)	1,100 (9.6%)	1,745 (14.0%)
II	228 (10.0%)	1,287 (24.6%)	839 (13.9%)	80 (4.6%)	241 (8.4%)	1,431 (12.4%)	792 (6.3%)
III	34 (1.5%)	194 (3.7%)	753 (12.5%)	132 (7.5%)	552 (19.2%)	834 (7.3%)	653 (5.2%)
IV	46 (2.0%)	61 (1.2%)	323 (5.3%)	119 (6.8%)	598 (20.8%)	762 (6.6%)	873 (7.0%)
Missing	1,533 (67.1%)	2,301 (43.9%)	3,607 (59.7%)	1,091 (62.1%)	858 (29.9%)	7,371 (64.1%)	8,437 (67.5%)
**Time between tumour diagnosis and AMI admission**							
Mean (SD)	5.3 (4.5)	6.4 (4.3)	5.2 (4.3)	4.6 (3.9)	2.4 (3.2)	5.4 (3.9)	4.7 (4.2)
Median (IQR)	4.2 (1.3-8.7)	6.0 (2.6-10.0)	4.4 (1.3-8.4)	3.5 (1.2-7.2)	1.0 (0.2-3.4)	4.6 (2.0-8.2)	3.6 (1.0-7.7)
**Laterality**							
Left	-	2,668 (51.0%)	-	756 (43.0%)	1,195 (41.6%)	-	-
Right	-	2,476 (47.3%)	-	850 (48.4%)	1,476 (51.4%)	-	-
Missing	-	92 (1.8%)	-	151 (8.6%)	199 (6.9%)	-	-
**Received Chemotherapy**	163 (39.2%)	117 (16.6%)	341 (29.7%)	34 (8.0%)	338 (31.1%)	52 (2.5%)	1,083 (38.3%)
**Received Radiotherapy**	104 (25.0%)	338 (47.9%)	161 (14.0%)	25 (5.9%)	299 (27.5%)	620 (29.6%)	588 (20.8%)
**Received Surgery**	240 (57.7%)	496 (70.3%)	863 (75.2%)	236 (55.3%)	270 (24.8%)	231 (11.0%)	533 (18.9%)
**Death following AMI admission**	**N=1,411**	**N=2,542**	**N=3,282**	**N=911**	**N=2,255**	**N=5,588**	**N=6,981**
Cancer	475 (33.7%)	610 (24.0%)	1,127 (34.3%)	370 (40.6%)	1,405 (62.3%)	1,712 (30.6%)	3,095 (44.3%)
CVD	556 (39.4%)	1,044 (41.1%)	1,222 (37.2%)	306 (33.6%)	480 (21.3%)	2,342 (41.9%)	2,350 (33.7%)
Other	271 (19.2%)	665 (26.2%)	678 (20.7%)	161 (17.7%)	306 (13.6%)	1,149 (20.6%)	1,164 (16.7%)
Missing	109 (7.7%)	223 (8.8%)	255 (7.8%)	74 (8.1%)	64 (2.8%)	385 (6.9%)	372 (5.3%)

Abbreviations: AMI, acute myocardial infarction; SD, standard deviation; IQR, interquartile range; CVD, cardiovascular disease.

Table S3. Missing data, n (%), in baseline characteristics of patients hospitalised with acute myocardial infarction, according to cancer history

	Cancer cases<br>N=42,187	Controls<br>N=470,201
Age at AMI admission*	0 (0.0%)	0 (0.0%)
Sex*	0 (0.0%)	0 (0.0%)
AMI phenotype†	0 (0.0%)	0 (0.0%)
Ethnicity	9,856 (30.5%)	116,406 (32.9%)
Previous AMI	3,551 (9.2%)	42,209 (9.9%)
Previous angina	3,889 (10.2%)	45,878 (10.8%)
Hypertension	3,437 (8.9%)	40,761 (9.5%)
High cholesterol	4,258 (11.2%)	48,146 (11.4%)
Cerebrovascular disease	3,894 (10.2%)	45,979 (10.8%)
Asthma/COPD	3,807 (9.9%)	45,200 (10.6%)
Chronic renal failure	3,996 (10.5%)	47,036 (11.1%)
Heart failure	3,897 (10.2%)	46,206 (10.9%)
Diabetes	1,304 (3.2%)	16,373 (3.6%)
Smoking status	3,479 (9.0%)	33,730 (7.7%)
Previous PCI	3,972 (10.4%)	45,993 (10.8%)
Previous CABG	3,865 (10.1%)	45,569 (10.7%)
Systolic blood pressure	4,138 (10.9%)	47,608 (11.3%)
Heart rate	4,148 (10.9%)	47,884 (11.3%)
Glucose	8,542 (25.4%)	89,664 (23.6%)
Creatinine	3,211 (8.2%)	37,680 (8.7%)
Haemoglobin	4,444 (11.8%)	52,284 (12.5%)
Beta blocker	5,929 (16.4%)	69,352 (17.3%)
ACE inhibitors or ARBs	5,925 (16.3%)	69,171 (17.2%)
Statins	3,238 (8.3%)	38,669 (9.0%)
Family history of CHD	11,072 (35.6%)	108,778 (30.1%)

*There is no missing information for patient age and sex as these variables were required for linkage between MINAP and NCRAS databases, patients with missing information were not included in the analysis.

†There is no missing information for AMI phenotype as this was required to identify AMI patients within the MINAP database, Non-AMI patients and those with missing phenotype information were not included in the analysis.

Abbreviations: AMI, acute myocardial infarction; COPD, chronic obstructive pulmonary disease; PCI, percutaneous coronary intervention; CABG, coronary artery bypass grafting; ACE, angiotensin-converting enzyme; ARB, angiotensin II receptor blockers; CHD, coronary heart disease.

Table S4. Baseline characteristics of patients hospitalised with acute myocardial infarction for cancer cases versus age-matched controls

	Cancer cases<br>N=42,187	Age-matched Controls<br>N=210,935	Absolute standardised difference
**Age at AMI admission**			
Mean (SD)	76.2 (10.2)	76.2 (10.2)	0.00016
Median (IQR)	77.3 (69.8-83.6)	77.2 (69.8-83.6)	
**Sex**			
Male	28,213 (66.9%)	126,796 (60.1%)	0.14085
Female	13,974 (33.1%)	84,139 (39.9%)	
**AMI phenotype**			
NSTEMI	28,779 (68.2%)	140,496 (66.6%)	0.03439
STEMI	13,408 (31.8%)	70,439 (33.4%)	
**Ethnicity**			
White	30,741 (95.1%)	146,270 (92.3%)	
Black	298 (0.9%)	1,408 (0.9%)	0.12946
Asian	885 (2.7%)	8,080 (5.1%)	
Mixed	55 (0.2%)	274 (0.2%)	
Other	352 (1.1%)	2,458 (1.6%)	
**Comorbidities**			
Previous AMI	8,470 (21.9%)	41,655 (21.6%)	0.00806
Previous angina	10,096 (26.4%)	49,749 (26.0%)	0.00815
Hypertension	20,995 (54.2%)	109,500 (56.5%)	0.04754
High cholesterol	11,341 (29.9%)	63,081 (33.2%)	0.07083
Cerebrovascular disease	3,895 (10.2%)	20,621 (10.8%)	0.01991
Asthma/COPD	6,972 (18.2%)	33,296 (17.4%)	0.02079
Chronic renal failure	3,822 (10.0%)	16,091 (8.4%)	0.05431
Heart failure	2,780 (7.3%)	13,515 (7.1%)	0.00724
Diabetes	9,864 (24.1%)	49,463 (24.2%)	0.00195
**Smoking status**			
Never smoked	10,878 (28.1%)	60,398 (31.1%)	
Ex-smoker	16,375 (42.3%)	72,635 (37.4%)	0.11108
Current smoker	5,922 (15.3%)	34,147 (17.6%)	
Non-smoker/unknown	5,533 (14.3%)	27,167 (14.0%)	
**Previous CVD procedures**			
Previous PCI	3,610 (9.4%)	18,102 (9.5%)	0.00081
Previous CABG	3,095 (8.1%)	15,128 (7.9%)	0.00647
**Systolic blood pressure**			
Mean (SD)	136.2 (28.3)	138.4 (28.8)	0.07457
Median (IQR)	135.0 (117.0-154.0)	137.0 (119.0-156.0)	
**Heart rate**			
Mean (SD)	82.2 (22.1)	81.2 (22.0)	0.04611
Median (IQR)	79.0 (67.0-94.0)	78.0 (66.0-92.0)	
**Glucose**			
Mean (SD)	8.4 (4.0)	8.4 (4.1)	0.00727
Median (IQR)	7.2 (6.0-9.4)	7.2 (6.0-9.4)	
**Creatinine**			
Mean (SD)	111.0 (74.6)	106.0 (65.8)	0.07101
Median (IQR)	93.0 (75.0-121.0)	91.0 (74.0-115.0)	
**Haemoglobin**			
Mean (SD)	125.3 (21.4)	130.5 (20.0)	0.25231
Median (IQR)	127.0 (111.0-140.0)	132.0 (118.0-144.0)	
**Medication prior to admission**			
Beta blocker	11,265 (31.1%)	54,812 (30.2%)	0.01787
ACE inhibitors or ARBs	13,952 (38.5%)	72,226 (39.9%)	0.02834
Statins	17,210 (44.2%)	87,109 (44.8%)	0.01148
**Family history of CHD**	6,318 (20.3%)	36,562 (23.1%)	0.06833

Abbreviations: AMI, acute myocardial infarction; SD, standard deviation; IQR, inter-quartile range; NSTEMI, non-ST-segment elevation myocardial infarction; STEMI, ST-segment elevation myocardial infarction; COPD, chronic obstructive pulmonary disease; CVD, cardiovascular disease; PCI, percutaneous coronary intervention; CABG, coronary artery bypass grafting; ACE, angiotensin-converting enzyme; ARB, angiotensin II receptor blockers; CHD, coronary heart disease.

Table S5. Early management and in-hospital treatment of patients admitted for AMI for cancer cases versus age-matched controls

STEMI: early management	Cancer cases<br>N=13,408	Age-Matched Controls<br>N=70,439	Absolute standardised difference
**Place ECG performed**			
Ambulance	9,215 (74.2%)	49,746 (76.4%)	0.06240
In hospital	2,593 (20.9%)	11,981 (18.4%)	
Other healthcare facility	610 (4.9%)	3,357 (5.2%)	
**First given aspirin/antiplatelet drug**			
Already on aspirin	1,902 (15.4%)	9,428 (14.5%)	0.09258
Given out of hospital	6,765 (54.7%)	37,998 (58.3%)	
Given after arrival in hospital	3,306 (26.7%)	16,345 (25.1%)	
Contraindicated	206 (1.7%)	683 (1.0%)	
Not given	186 (1.5%)	667 (1.0%)	
**Anticoagulant**			
Fondaparinux	1,732 (16.8%)	9,065 (16.8%)	0.00196
Unfractionated heparin	4,557 (44.2%)	25,009 (46.3%)	0.04095
Low molecular weight heparin	4,654 (44.8%)	23,748 (43.6%)	0.02543
**Thrombolytic treatment**	406 (3.0%)	2,709 (3.9%)	0.04497
**Angiogram**	10,956 (81.7%)	60,346 (85.7%)	0.08641
**Percutaneous Coronary Intervention**	9,634 (71.9%)	53,566 (76.1%)	0.09609
**CABG**	86 (0.9%)	813 (1.6%)	0.06232
**NSTEMI: early management**	**Cancer cases<br>N=28,779**	**Age-Matched Controls<br>N=140,496**	**Absolute standardised difference**
**Place ECG performed**			
Ambulance	13,416 (51.6%)	66,437 (52.3%)	0.03533
In hospital	11,878 (45.7%)	56,581 (44.5%)	
Other healthcare facility	692 (2.7%)	4,018 (3.2%)	
**First given aspirin/antiplatelet drug**			
Already on aspirin	7,919 (29.6%)	39,513 (30.1%)	0.05274
Given out of hospital	5,698 (21.3%)	29,546 (22.5%)	
Given after arrival in hospital	11,858 (44.3%)	56,878 (43.3%)	
Contraindicated	879 (3.3%)	3,426 (2.6%)	
Not given	440 (1.6%)	1,897 (1.4%)	
**Anticoagulant**			
Fondaparinux	11,746 (48.5%)	58,619 (49.3%)	0.01446
Unfractionated heparin	3,178 (13.3%)	15,674 (13.3%)	0.00186
Low molecular weight heparin	12,314 (51.1%)	60,735 (51.3%)	0.00280
**Thrombolytic treatment**	45 (0.2%)	187 (0.1%)	0.00620
**Angiogram**	15,629 (54.5%)	83,639 (59.8%)	0.10584
**Percutaneous Coronary Intervention**	7,466 (26.1%)	39,278 (28.1%)	0.04545
**CABG**	637 (3.0%)	4,206 (4.0%)	0.05308

Abbreviations: AMI, acute myocardial infarction; STEMI, ST-segment elevation myocardial infarction; ECG, electrocardiogram; CABG, coronary artery bypass graft; NSTEMI, non-ST-segment elevation myocardial infarction.

Table S6. Eligible for quality indicator assessment

Quality Indicator		Cancer cases	Age-matched Controls
	**STEMI patients**	**N=13,408**	**N=70,439**
**2.1:** Reperfusion within 12h of presentation	Not eligible	1,472 (11.0%)	7,098 (10.1%)
	Eligible	8,782 (65.5%)	48,674 (69.1%)
	Cannot be assessed	3,154 (23.5%)	14,667 (20.8%)
**2.2:** Timely reperfusion	Not eligible	2,784 (20.8%)	13,840 (19.6%)
	Eligible	7,261 (54.2%)	40,812 (57.9%)
	Cannot be assessed	3,363 (25.1%)	15,787 (22.4%)
**2.4:** Time from diagnosis to wire passage	Not eligible	2,772 (20.7%)	13,999 (19.9%)
	Eligible	7,460 (55.6%)	41,664 (59.1%)
	Cannot be assessed	3,176 (23.7%)	14,776 (21.0%)
	**NSTEMI patients**	**N=28,779**	**N=140,496**
**2.3:** Coronary angiography received within 72h	Not eligible	8,329 (28.9%)	43,237 (30.8%)
	Eligible	10,479 (36.4%)	54,303 (38.7%)
	Cannot be assessed	9,971 (34.6%)	42,956 (30.6%)
**4.2:** Fondaparinux received	Not eligible	970 (3.4%)	3,940 (2.8%)
	Eligible	27,809 (96.6%)	136,556 (97.2%)
	**All patients**	**N=42,187**	**N=210,935**
**3.3:** LV function recorded in notes	Eligible	42,187 (100.0%)	210,935 (100.0%)
**4.1:** Adequate $P2Y_{12}$ inhibition on discharge	Not eligible	8,392 (19.9%)	37,069 (17.6%)
	Eligible	32,232 (76.4%)	166,939 (79.1%)
	Cannot be assessed	1,563 (3.7%)	6,927 (3.3%)
**4.3:** DAPT received on discharge	Not eligible	6,649 (15.8%)	30,499 (14.5%)
	Eligible	30,460 (72.2%)	159,132 (75.4%)
	Cannot be assessed	5,078 (12.0%)	21,304 (10.1%)
**5.1:** High intensity statins on discharge	Not eligible	12,525 (29.7%)	58,283 (27.6%)
	Eligible	29,539 (70.0%)	152,110 (72.1%)
	Cannot be assessed	123 (0.3%)	542 (0.3%)
**5.2:** ACEi/ARB on discharge for those with HF or LVEF≤50	Not eligible	26,241 (62.2%)	129,962 (61.6%)
	Eligible	13,022 (30.9%)	66,034 (31.3%)
	Cannot be assessed	2,924 (6.9%)	14,939 (7.1%)
**5.3:** β-blocker on discharge for those with HF or LVEF≤50	Not eligible	26,195 (62.1%)	129,626 (61.5%)
	Eligible	13,146 (31.2%)	66,678 (31.6%)
	Cannot be assessed	2,846 (6.7%)	14,631 (6.9%)
**7.1:** Composite QI (opportunity-based)	Not eligible	3,685 (8.7%)	15,713 (7.4%)
	Eligible	36,857 (87.4%)	187,516 (88.9%)
	Cannot be assessed	1,645 (3.9%)	7,706 (3.7%)
**7.2:** Composite QI (all-or-none, overall score)	Eligible	39,615 (93.9%)	198,770 (94.2%)
	Cannot be assessed	2,572 (6.1%)	12,165 (5.8%)

Abbreviations: STEMI, ST-segment elevation myocardial infarction; NSTEMI, non-ST-segment elevation myocardial infarction; LV, left ventricular; DAPT, dual antiplatelet therapy; ACEi, angiotensin-converting enzyme inhibitors; ARB, angiotensin receptor blockers; HF, heart failure; LVEF, left ventricular ejection fraction; QI, quality indicator.

Table S7. Comparison of different standard error selection when assessing differences in quality indicator attainment

Quality Indicator	Difference PP receiving care, with robust standard errors, (95% CI)*			Difference PP receiving care, with hospital clustered standard errors, (95% CI)*		
	Unadjusted	Adjusted†	p-value	Unadjusted	Adjusted†	p-value
**2.1:** Reperfusion within 12h of presentation (STEMI)	-2.7% (-3.5, -1.8)	-0.5% (-1.3, 0.4)	0.26	-2.7% (-3.6, -1.8)	-0.5% (-1.4, 0.4)	0.26
**2.2:** Timely reperfusion (STEMI)	-0.7% (-1.8, 0.4)	0.6% (-0.5, 1.6)	0.32	-0.7% (-1.8, 0.5)	0.6% (-0.5, 1.6)	0.31
**2.3:** Coronary angiography received within 72h (NSTEMI)	-5.2% (-6.7, -3.6)	-0.9% (-2.4, 0.6)	0.25	-5.2% (-6.8, -3.6)	-0.9% (-2.5, 0.7)	0.26
**2.4:** Time from diagnosis to wire passage (STEMI): mins, med [IQR]‡	1.6 (-1.0, 4.2)	0.3 (-2.4, 2.9)	0.83	1.6 (-0.8, 4.0)	0.3 (-1.9, 2.4)	0.80
**3.3:** LV function recorded in notes||	-2.2% (-2.8, -1.7)	-0.9% (-1.5, -0.4)	$8.0e^{-4}$	-2.2% (-3.2, -1.3)	-0.9% (-1.5, -0.3)	$3.5e^{-3}$
**4.1:** Adequate $P2Y_{12}$ inhibition on discharge	-0.4% (-0.5, -0.3)	-0.3% (-0.4, -0.1)	$1.0e^{-5}$	-0.4% (-0.5, -0.3)	-0.3% (-0.4, -0.1)	$1.95e^{-5}$
**4.2:** Fondaparinux received (NSTEMI)	-2.1% (-2.8, -1.5)	-0.4% (-1.1, 0.2)	0.21	-2.1% (-3.1, -1.1)	-0.4% (-1.3, 0.4)	0.30
**4.3:** DAPT received on discharge	-0.5% (-0.6, -0.3)	-0.3% (-0.4, -0.1)	$4.0e^{-5}$	-0.5% (-0.6, -0.3)	-0.3% (-0.4, -0.2)	$1.09e^{-5}$
**5.1:** High intensity statins on discharge	-1.3% (-1.6, -1.1)	-0.8% (-1.0, -0.6)	$9.4e^{-12}$	-1.3% (-1.6, -1.1)	-0.8% (-1.0, -0.5)	$1.71e^{-9}$
**5.2:** ACEi/ARB on discharge for those with HF or LVEF≤50	-6.3% (-7.1, -5.5)	-2.6% (-3.4, -1.8)	$2.9e^{-10}$	-6.3% (-7.3, -5.3)	-2.6% (-3.5, -1.7)	$6.51e^{-9}$
**5.3:** β-blocker on discharge for those with HF or LVEF≤50	-3.4% (-4.2, -2.6)	-0.6% (-1.4, 0.2)	0.14	-3.4% (-4.4, -2.3)	-0.6% (-1.5, 0.3)	0.20
**7.1:** Composite QI (opportunity-based): Mean (SD)#	-1.6% (-1.8, -1.4)	-0.5% (-0.7, -0.4)	$5.1e^{-10}$	-1.6% (-1.9, -1.3)	-0.5% (-0.7, -0.3)	$3.09e^{-7}$
**7.2:** Composite QI (all-or-none, overall score)#	-4.1% (-4.5, -3.7)	-1.2% (-1.6, -0.9)	$2.5e^{-11}$	-4.1% (-4.6, -3.6)	-1.2% (-1.6, -0.8)	$1.12e^{-9}$

*The reported difference is the difference between the observed proportion of cancer patients who received care and the counterfactual proportion of cancer patients who would have received care had they not had a previous cancer diagnosis, i.e. the average effect of cancer on quality indicator attainment in the cancer population (average treatment effect of the treated).

†Adjusted for age (non-linear), sex, AMI phenotype, comorbidities (previous AMI, angina, hypertension, hypercholesterolaemia, cerebrovascular, asthma, COPD, chronic renal failure, heart failure, diabetes), smoking status, and previous CVD procedures (percutaneous coronary intervention, coronary artery bypass graft)

‡Difference in mean time from diagnosis to wire passage is reported for quality indicator 2.4, rather than difference in proportion.

||Can only assess if LVEF category recorded, not numerical value

#Composite quality indicators summarize information from different domains into a single measure. The opportunity-based score assesses the following QIs where the patient is eligible; 2.1, 2.3, 3.3, 4.1, 5.1, 5.2, 5.3, and is calculated as total receiving care out of total eligible. The all-or-none score assesses 3 components in patients without heart failure and LVEF>0.50 (low dose aspirin and QIs 4.1, 5.1) and 5 in patients with heart failure or LVEF≤50 (low dose aspirin and QIs 4.1, 5.1, 5.2, 5.3), calculated as the proportion of patients who achieve all of components.

Abbreviations: PP, percentage points; CI, confidence interval; STEMI, ST-segment elevation myocardial infarction; NSTEMI, non-ST elevation myocardial infarction; IQR, interquartile range; LV, left ventricular; DAPT, dual antiplatelet therapy; ACEi, angiotensin-converting enzyme inhibitors; ARB, angiotensin receptor blockers; HF, heart failure; LVEF, left ventricular ejection fraction; QI, quality indicator; SD, standard deviation.

Table S8. In-hospital mortality following AMI admission in cancer cases, controls and counterfactual (adjusted) controls

Cancer cases % (95% CI)	Controls % (95% CI)	Counterfactual Controls (adjusted)	Difference in percentage who die in-hospital	
			Unadjusted	Adjusted
7.9% (7.6, 8.1)	4.8% (4.8, 4.9)	6.5% (6.4, 6.6)	3.0% (2.7, 3.3)	1.4% (1.1, 1.7)

Abbreviations: CI, confidence interval

The RECORD statement – checklist of items, extended from the STROBE statement, that should be reported in observational studies using routinely collected health data.

	Item No.	STROBE items	Location in manuscript where items are reported	RECORD items	Location in manuscript where items are reported
**Title and abstract**					
	1	(a) Indicate the study's design with a commonly used term in the title or the abstract (b) Provide in the abstract an informative and balanced summary of what was done and what was found	Title	RECORD 1.1: The type of data used should be specified in the title or abstract. When possible, the name of the databases used should be included.<br><br>RECORD 1.2: If applicable, the geographic region and timeframe within which the study took place should be reported in the title or abstract.<br><br>RECORD 1.3: If linkage between databases was conducted for the study, this should be clearly stated in the title or abstract.	Abstract (Methods)<br><br>Abstract (Methods)<br><br>Title<br>Abstract (Methods)
**Introduction**					
Background rationale	2	Explain the scientific background and rationale for the investigation being reported	Introduction – paragraphs 1-3		
Objectives	3	State specific objectives, including any prespecified hypotheses	Introduction – paragraph 4		
**Methods**					
Study Design	4	Present key elements of study design early in the paper	Methods (Study population) – paragraphs 1-2		
Setting	5	Describe the setting, locations, and	Methods (Study		

		relevant dates, including periods of recruitment, exposure, follow-up, and data collection	population):<br>Para2 - setting/location/date<br>Para3-4 – exposure<br>Para5-6 - outcome		
Participants	6	*(a) Cohort study* - Give the eligibility criteria, and the sources and methods of selection of participants. Describe methods of follow-up<br>*Case-control study* - Give the eligibility criteria, and the sources and methods of case ascertainment and control selection. Give the rationale for the choice of cases and controls<br>*Cross-sectional study* - Give the eligibility criteria, and the sources and methods of selection of participants<br><br>*(b) Cohort study* - For matched studies, give matching criteria and number of exposed and unexposed<br>*Case-control study* - For matched studies, give matching criteria and the number of controls per case	Methods (Study population): paragraph 2 Supplementary methods.<br><br>Methods (Statistical analysis): paragraph 1	RECORD 6.1: The methods of study population selection (such as codes or algorithms used to identify subjects) should be listed in detail. If this is not possible, an explanation should be provided.<br><br>RECORD 6.2: Any validation studies of the codes or algorithms used to select the population should be referenced. If validation was conducted for this study and not published elsewhere, detailed methods and results should be provided.<br><br>RECORD 6.3: If the study involved linkage of databases, consider use of a flow diagram or other graphical display to demonstrate the data linkage process, including the number of individuals with linked data at each stage.	Methods (Study population): paragraph 2 Supplementary methods. Supplementary figure S1.<br>Supplementary methods. Supplementary figure S1.<br><br>NA – Data resource profile referenced (ref 13) containing information
Variables	7	Clearly define all outcomes, exposures, predictors, potential confounders, and effect modifiers. Give diagnostic criteria, if applicable.	Methods: (Evidence-based hospital treatment): para1-2 – outcome (Study population):	RECORD 7.1: A complete list of codes and algorithms used to classify exposures, outcomes, confounders, and effect modifiers should be provided. If these cannot be reported, an explanation	Methods (Evidence-based hospital treatment): para1-2

			para3-4 – exposure (Statistical analysis): para2-3 – predictors, confounders, moderators	should be provided.	Supplementary Table S1. Supplementary figure S1.
Data sources/ measurement	8	For each variable of interest, give sources of data and details of methods of assessment (measurement).<br>Describe comparability of assessment methods if there is more than one group	Methods (Evidence-based hospital treatment): para1-2 – outcome (Study population): para3-4 – exposure (Statistical analysis): para2-3 – predictors, confounders, moderators		
Bias	9	Describe any efforts to address potential sources of bias	Methods (Statistical analysis): paragraph 1-2		
Study size	10	Explain how the study size was arrived at	Methods (Study population): paragraph 1-2		
Quantitative variables	11	Explain how quantitative variables were handled in the analyses. If applicable, describe which groupings were chosen, and why	Supplementary methods.		
Statistical methods	12	(a) Describe all statistical methods, including those used to control for confounding<br>(b) Describe any methods used to examine subgroups and interactions<br>(c) Explain how missing data were	Methods (Statistical analysis): paragraph 1-4<br>Supplementary methods.		

		addressed<br>(d) *Cohort study* - If applicable, explain how loss to follow-up was addressed<br>*Case-control study* - If applicable, explain how matching of cases and controls was addressed<br>*Cross-sectional study* - If applicable, describe analytical methods taking account of sampling strategy<br>(e) Describe any sensitivity analyses			
Data access and cleaning methods		..		RECORD 12.1: Authors should describe the extent to which the investigators had access to the database population used to create the study population.<br><br>RECORD 12.2: Authors should provide information on the data cleaning methods used in the study.	Methods (Study population): paragraph 1<br><br>Data resource profile referenced (ref 13) containing information
Linkage		..		RECORD 12.3: State whether the study included person-level, institutional-level, or other data linkage across two or more databases. The methods of linkage and methods of linkage quality evaluation should be provided.	Methods (Study population): paragraph 1-2
**Results**					
Participants	13	(a) Report the numbers of individuals at each stage of the study (*e.g.*, numbers potentially	Results (Study population): paragraph 1	RECORD 13.1: Describe in detail the selection of the persons included in the study (*i.e.,* study population selection)	Methods (Study population): paragraph 1-2

		eligible, examined for eligibility, confirmed eligible, included in the study, completing follow-up, and analysed)<br>(b) Give reasons for non-participation at each stage.<br>(c) Consider use of a flow diagram	Tables 1-3	including filtering based on data quality, data availability and linkage. The selection of included persons can be described in the text and/or by means of the study flow diagram.	Results (Study population): paragraph 1
Descriptive data	14	(a) Give characteristics of study participants (*e.g.*, demographic, clinical, social) and information on exposures and potential confounders<br>(b) Indicate the number of participants with missing data for each variable of interest<br>(c) *Cohort study* - summarise follow-up time (*e.g.*, average and total amount)	Results (Study population): paragraph 1&3 Table 1.<br><br>Results (Study population): paragraph 2		
Outcome data	15	*Cohort study* - Report numbers of outcome events or summary measures over time<br>*Case-control study* - Report numbers in each exposure category, or summary measures of exposure<br>*Cross-sectional study* - Report numbers of outcome events or summary measures	Table 3		
Main results	16	(a) Give unadjusted estimates and, if applicable, confounder-adjusted estimates and their precision (e.g., 95% confidence interval). Make clear which confounders were	Table 3<br>Figure 1		

		adjusted for and why they were included<br>(b) Report category boundaries when continuous variables were categorized<br>(c) If relevant, consider translating estimates of relative risk into absolute risk for a meaningful time period	 <br>NA<br><br><br>Table 3		
Other analyses	17	Report other analyses done—e.g., analyses of subgroups and interactions, and sensitivity analyses	Figure 3,4&5<br>Supplementary Table S4,S7<br>Figure S2,S3,S4		
**Discussion**					
Key results	18	Summarise key results with reference to study objectives	Discussion – paragraph 1		
Limitations	19	Discuss limitations of the study, taking into account sources of potential bias or imprecision. Discuss both direction and magnitude of any potential bias	Discussion (Limitations)	RECORD 19.1: Discuss the implications of using data that were not created or collected to answer the specific research question(s). Include discussion of misclassification bias, unmeasured confounding, missing data, and changing eligibility over time, as they pertain to the study being reported.	Discussion (Limitations): paragraph 1
Interpretation	20	Give a cautious overall interpretation of results considering objectives, limitations, multiplicity of analyses, results from similar studies, and other relevant evidence	Discussion (Conclusions)		
Generalisability	21	Discuss the generalisability (external validity) of the study results	Discussion (Limitations)		

Other Information					
Funding	22	Give the source of funding and the role of the funders for the present study and, if applicable, for the original study on which the present article is based	Abstract (Funding)<br>End matter (Funding)		
Accessibility of protocol, raw data, and programming code		..		RECORD 22.1: Authors should provide information on how to access any supplemental information such as the study protocol, raw data, or programming code.	Methods (Data disclosure)

Reference: Benchimol EI, Smeeth L, Guttmann A, Harron K, Moher D, Petersen I, Sørensen HT, von Elm E, Langan SM, the RECORD Working Committee. The REporting of studies Conducted using Observational Routinely-collected health Data (RECORD) Statement. *PLoS Medicine* 2015; in press.

Checklist is protected under Creative Commons Attribution (CC BY) license.
